# Appraisal of Non-Cardiovascular Safety for Sodium–Glucose Co-Transporter 2 Inhibitors: A Systematic Review and Meta-Analysis of Placebo-Controlled Randomized Clinical Trials

**DOI:** 10.3389/fphar.2019.01066

**Published:** 2019-09-19

**Authors:** Fang-Hong Shi, Hao Li, Long Shen, Zhen Zhang, Yi-Hong Jiang, Yao-Min Hu, Xiao-Yan Liu, Zhi-Chun Gu, Jing Ma, Hou-Wen Lin

**Affiliations:** ^1^Department of Pharmacy, Renji Hospital, School of Medicine, Shanghai Jiao Tong University, Shanghai, China; ^2^Department of Pharmacy, Shanghai Children’s Medical Center, School of Medicine, Shanghai Jiao Tong University, Shanghai, China; ^3^Department of Cardiology, Renji Hospital, School of Medicine, Shanghai Jiao Tong University, Shanghai, China; ^4^Pharmacy Department, Memorial Healthcare System, Hollywood, FL, United States; ^5^Department of Endocrinology and Metabolism, Renji Hospital, School of Medicine, Shanghai Jiao Tong University, Shanghai, China

**Keywords:** type 2 diabetes mellitus, sodium-glucose co-transporter 2 inhibitors, non-cardiovascular safety, randomized controlled trials, systematic review, meta-analysis

## Abstract

**Background:** Whereas the cardiovascular safety of sodium-glucose co-transporter 2 (SGLT2) inhibitors has been well reported, there is limited data from controlled clinical trials regarding the non-cardiovascular safety. This was the focus of our study.

**Methods and Findings:** We systematically searched MEDLINE, EMBASE, and Cochrane Library (5^th^ Sep 2018) for randomized controlled trials (RCTs) that reported safety data for SGLT2 inhibitors and placebo. Relative risks (RRs) and their 95% confidence intervals (CIs) were pooled using random-effects models. Seventy RCTs (83 studies enrolling 36,958 patients in 78 publications) were identified. SGLT2 inhibitors were associated with a lower risk of serious adverse events (RR 0.90, 95% CI 0.86 to 0.94, *P* < 0.001), death (RR 0.78, 95% CI 0.64 to 0.94, *P* < 0.05), gastroenteritis (RR 0.38, 95% CI 0.20 to 0.72, *P* < 0.05), arthralgia (RR 0.72, 95% CI 0.54 to 0.96, *P* < 0.05), hypertension (RR 0.61, 95% CI 0.50 to 0.75, *P* < 0.001), and edema/peripheral edema (RR 0.49, 95% CI 0.33 to 0.72, *P* < 0.001) compared to placebo. SGLT2 inhibitors were associated with higher risk of infections compared to placebo (RR 1.27, 95% CI 1.17 to 1.37, *P* < 0.001), especially for genital mycotic infection (GMI) (RR 3.71, 95% CI 3.19 to 4.32, *P* < 0.001). Other significant effects were observed for osmotic diuresis–related AEs (RR 2.73, 95% CI 2.20 to 3.40, *P* < 0.001), volume-related AEs (RR 1.26, 95% CI 1.08 to 1.46, *P* < 0.05), renal-related AEs (RR 1.36, 95% CI 1.02 to 1.80, *P* < 0.05), hypoglycemia (RR 1.18, 95% CI 1.10 to 1.26, *P* < 0.001), and increased blood ketone bodies (RR 2.00, 95% CI 1.01 to 3.97, *P* < 0.05). Subgroup and sensitivity analyses strengthened the robustness of primary results.

**Conclusion:** Results from RCTs confirmed lower risk of death, serious adverse events, hypertension, and edema associated with type 2 diabetes mellitus (T2DM) patients treated with SGLT2 inhibitors when compared with placebo. The use of SGLT2 inhibitors were associated with higher risk of infection, osmotic diuresis, volume depletion effects, renal related AEs, and higher blood ketone bodies when compared with placebo.

## Introduction

Type 2 diabetes mellitus (T2DM) is a complex disease characterized with hyperglycemia and progressive dysregulation of insulin–glucose feedback mechanisms (Defronzo, 2009). It is the sixth leading cause of disability (GBD 2015 Disease and Injury Incidence and Prevalence Collaborators, 2016; Chatterjee et al., 2017). The prevalence of T2DM is rapidly increasing especially in developing countries (King et al., 1998; Bommer et al., 2018). In Asia, patients with T2DM without glucose management have higher risk of complications such as cardiovascular disease (coronary heart disease, stroke, and peripheral vascular disease), renal disease (chronic kidney disease), obesity, inflammation, glucolipotoxicity, and oxidative stress (Kong et al., 2013; Chatterjee et al., 2017). Guidelines of the American Diabetes Association (ADA) in 2018 recommended metformin as first-line pharmacological therapy in T2DM (American Diabetes Association). The rest of the hypoglycemic agents are used as second-line treatment option when glycemic control is not achieved with metformin, or unable to tolerate metformin due to gastrointestinal side-effects or contraindications (Defronzo, 2009).

Sodium–glucose co-transporter 2 (SGLT2) inhibitors (also termed gliflozins) are a well-tolerated, newly approved oral hypoglycemic drug, capable of lowering glucose by reducing renal glucose reabsorption and causing urinary glucose excretion, providing a new second-line choice for the treatment of T2DM (Tsimihodimos et al., 2018). Previous studies emphasized the glucose lowering effects of SGLT2 inhibitors but often ignored their safety issues. With the widespread clinical use, the adverse effects, such as infection-related (Li et al., 2017; Liu et al., 2017; Puckrin et al., 2018) or renal-related adverse events (Perlman et al., 2017; Tang et al., 2017; Xu et al., 2017), had been raised. Meanwhile, the US Food and Drug Administration (FDA) had issued warnings about the occurrences of serious infection of the genitals called necrotizing fasciitis, serious urinary tract infections and ketoacidosis for all SGLT2 inhibitors, acute kidney injury for canagliflozin and dapagliflozin, risk of leg or foot amputations, risk of bone fractures, and risk of bone fractures for canagliflozin during post-marketing studies of SGLT2 inhibitors (FDA, 2015a; FDA, 2015b; FDA, 2016; FDA, 2017; FDA, 2018). A prior meta-analysis had suggested cardiovascular protection and safety outcomes of SGLT2 inhibitors. However, the non-cardiovascular safety results were imprecise and not given a systematic and comprehensive evaluation (Radholm et al., 2018). The aim of this study was to conduct a systematic review to identify yet unknown adverse drug effects of SGLT2 inhibitors, and where possible, perform meta-analyses to compare the reported effects between SGLT2 inhibitors and a placebo comparator.

## Methods

### Study Design

We followed *a priori* established protocol (PROSPERO: CRD42018090153) and the standards in Cochrane Collaboration and PRISMA guidelines for reporting a meta-analysis (Shamseer et al., 2015; Li et al., 2018). MEDLINE, Embase, and the Cochrane library were searched for eligible trials up to 5^th^ Sep, 2018. We selected medical subject heading (MeSH) terms and free-text terms relating to individual gliflozin. For the topic of “type 2 diabetes,” the following key terms were used for searching: “type 2 diabetes” and “type 2 diabetes mellitus.” For the topic of “SGLT2 inhibitors,” we included the following terms: “sodium-glucose co-transporter 2 inhibitors,” “SGLT2 inhibitors,” “SGLT2,” and both generic and trade names of each SGLT2 inhibitors. For the topic of “randomized controlled trials (RCTs),” the terms used were: “clinical trial” and “controlled clinical trial” and “randomized controlled trial.” We used the Boolean operator “AND” to combine the three comprehensive search themes. Additionally, a manual search of the “ClinicalTrials.gov” website for the retrieved references, relevant meta-analyses, and reviews were carried out to identify additional trials. The detailed search strategy was shown in **Table S1**. Three reviewers (F-HS and HL and LS) independently evaluated the eligibility of each relevant study, and any disagreements were resolved by consulting corresponding author (Z-CG).

### Inclusion Criteria and Study Selection

Studies meeting the following criteria were included: (1) studies, double-blinded RCTs; (2) population, adults’ patients with T2DM; (3) intervention, SGLT2 inhibitors monotherapy or as add-on to other hypoglycemic therapy; (4) comparison, placebo; (5) outcome, any clinical adverse events, overall adverse events (AEs), and AEs occurring in ≥3% patients; (6) follow-up duration, at least 12 weeks; and (7) samples, at least 100 patients. We excluded observational studies, pooled analyses, and trials that were not randomized or used the control as active therapy or standard care.

### Data Extraction

Full-text articles along with accessible supplementary materials from eligible publications were retrieved for data extraction. The following information was extracted independently by three investigators (F-HS and HL and LS): first author’s name, year of publication, number of study patients, baseline patient characteristics, intervention (type of SGLT2 inhibitors and corresponding dose), comparison (placebo), follow-up duration, and safety outcome (occurrence number and total number). While our research is still in progress, several cardiovascular protection and safety outcomes of SGLT2 inhibitors had been reported (Tang et al., 2016; Wu et al., 2016; Radholm et al., 2018; Usman et al., 2018; Zhang et al., 2018). In this study, we only reported non-cardiovascular safety outcomes of SGLT2 inhibitors.

### Quality Assessment and Bias Assessment

Three reviewers (F-HS and HL and LS) evaluated the methodological quality of included RCTs according to the Cochrane Collaboration Risk of Bias Tool, which included random sequence generation, allocation concealment, masking, incomplete outcome data, selective reporting, and other bias. Any disagreement was settled by discussing with corresponding author (Z-CG).

### Data Analysis

The estimates of meta-analysis were derived and presented in forest plots by using STATA version 12.0 (STATA Corporation, College Station, TX, USA). Continuous variables expressed as weight mean difference (WMD) with their 95% confidence intervals (95% CIs), and dichotomous data were reported as relative risk (RRs) with 95% CIs. The random-effect model was used to calculate the overall estimated effects. Heterogeneity among studies was explored using the *I* ^2^ statistic (significance for *I*2 > 50%) (Higgins and Thompson, 2002). Prespecified subgroup analyses were conducted according to individual SGLT2 inhibitors (7 SGLT2 inhibitors), durations of follow-up, and monotherapy or not. Leave-one-out studies were used for sensitivity analysis. Researches with two follow-up times were considered as independent studies when these results were published separately. Further leave-out studies were used to test the consistency with primacy analyses by excluding the studies with short follow-ups. Potential publication bias was evaluated by visual funnel plots as well as quantitative Begg’s test and Egger’s test. *P* < 0.05 indicated a statistically significant difference.

## Results

### Study Selection and Characteristics

Of 9,412 records identified by initially electronic search, 78 relevant publications (83 paired comparisons, 7 SGLT2 inhibitors) that met our inclusion criteria were identified (**Figure S1**). The sample size ranged from 129 to 7,020, totaling 36,958 patients with T2DM.

Characteristics of the included trials were summarized in **Table S2**. In total, 36,958 participants were included in 83 comparisons (13 researches had two different follow-up duration and safety results, which were considered as 26 separated studies). Among these participants, 29,095 T2DM patients received different kinds of SGLT2 inhibitors (empagliflozin, canagliflozin, dapagliflozin, tofogliflozin, luseogliflozin, ipragliflozin, or ertugliflozin). Publication year varied from 2009 to 2018, and the trial duration ranged from 12 to 161 weeks. Among 78 publications, 28 publications enrolled from Asians, 43 publications from Caucasians, and the remaining 7 publications were unclear. Participants were generally middle-aged (mean age was 58 years old). The mean HbA1C% was 8.1%, mean body mass index (BMI) was 29.4 kg/m^2^, and the mean duration of diabetes was 7.9 years.

### Risk of Bias

All 83 studies included in this meta-analysis were double-blinded, placebo-controlled RCTs with randomized sequence generation and allocation concealment. Among these studies, 15 studies contained both double-blinded and open-label data. However, only double-blinded data were included in this study. The majority of included studies were judged to have a low risk of bias. Details of included studies were presented in **Table S3**.

### Safety Outcomes

#### Overall Safety

**Figure 1:**
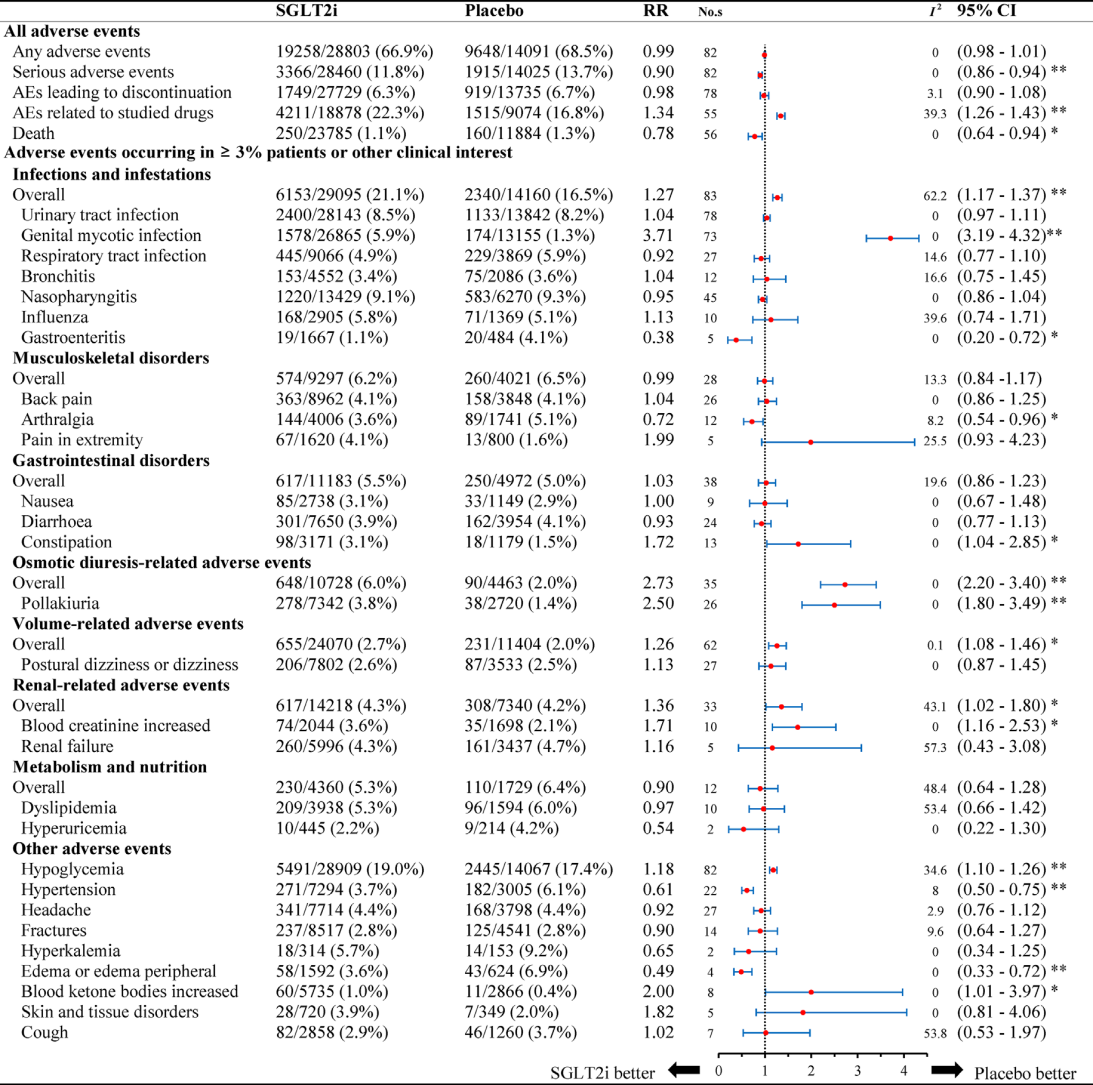
Relative risk of adverse events reported for SGLT2i in comparison to placebo. Abbreviations: No.s, number of studies. * *P* < 0.05, ** *P* < 0.001.

Overall safety outcomes include any adverse events, serious adverse event, AEs leading to discontinuation, AEs related to studied drugs, and AEs associated with death (**Figure 1**). The incidence of death from any cause was 1.1% (250/23,785) in the SGLT2 inhibitors group, while it was 1.3% (160/11,884) in the placebo group. In addition, the incidence of serious adverse events was 11.8% (3,366/28,460) in SGLT2 inhibitors and 13.7% (1,915/14,025) in placebo group. SGLT2 inhibitors were associated with a lower risk of death (RR: 0.78, 95% CI: 0.64 to 0.94, *P* < 0.05) and serious AEs (RR: 0.90, 95% CI: 0.86 to 0.94, *P* < 0.001) (**Figure 1**). SGLT2 inhibitors might not increase any AEs risk. (RR: 0.99, 95% CI: 0.98 to 1.01, *P* = 0.313) and AEs leading to premature treatment discontinuation (RR: 0.98, 95% CI: 0.90 to 1.08, *P* = 0.809), with the exception of AEs related to studied drugs (RR: 1.34, 95% CI: 1.26 to 1.43, *P* < 0.001). No significant heterogeneity was observed among included studies (*I* ^2^ = 0% for any adverse events, *I* ^2^ = 0 for serious adverse events, *I* ^2^ = 3.1% for AEs leading to discontinuation, *I* ^2^ = 39.3% for AEs related to studied drugs, *I* ^2^ = 0 for death).

#### Specified Safety

**Figure 1** summarizes the AEs events occurring in ≥ 3% patients and other 35 clinically interest safety items. For infections, the incidence rate was 21.1% (6,153/29,095) in the SGLT2 inhibitors group, while it was 16.5% (2,340/14,160) in the placebo group. SGLT2 inhibitors were associated with a higher risk of infections compared to placebo (RR 1.27, 95% CI: 1.17 to 1.37, *P* < 0.001), especially for genital mycotic infection (GMI) (RR 3.71, 95% CI: 3.19 to 4.32, *P* < 0.001). In patients with gastroenteritis, the use of SGLT2 inhibitors was associated with a lower risk as compared to placebo (RR 0.38, 95% CI: 0.20 to 0.72, *P* < 0.05). There was no significant difference in patients with risk of urinary tract infection (UTI), respiratory tract infection, bronchitis, nasopharyngitis, and influenza. No significant heterogeneity was observed for each infection site (*I* ^2^ = 0% for UTI, *I* ^2^ = 0 for GMI, *I* ^2^ = 14.6% for respiratory tract infection, *I* ^2^ = 16.6% for bronchitis, *I* ^2^ = 0 for nasopharyngitis, *I* ^2^ = 39.6% for influenza, and *I* ^2^ = 0 for gastroenteritis).

For other adverse events, the risk of osmotic diuresis-related AEs was higher in patients with SGLT2 inhibitors *versus* placebo (RR 2.73, 95% CI: 2.20 to 3.40, *P* < 0.001). The consistent result was observed for pollakiuria (RR 2.50, 95% CI 1.80 to 3.49, *P* < 0.001). As for volume-related AEs, SGLT2 inhibitors also associated with a higher risk (RR 1.26, 95% CI: 1.08 to1.46, P < 0.05). Additionally, other significant effects were observed for renal-related AEs (RR 1.36, 95% CI 1.02 to 1.80, *P* < 0.05). No significant heterogeneity was detected among included studies (*I* ^2^ = 0% for osmotic diuresis–related AEs, *I* ^2^ = 0 for pollakiuria, *I* ^2^ = 0.1% for volume-related AEs, *I* ^2^ = 43.1% for renal-related AEs, *I* ^2^ = 0 for skin and tissue disorders).

SGLT2 inhibitors were associated with a higher risk of abnormal blood ketone bodies as compared to placebo (1.0% *vs*. 0.4%), with a corresponding RR of 2.00 (95% CI: 1.01 to 3.97, *P* < 0.05). Additionally, total incidence of hypoglycemia was 19.0% (5,491/28,909) in patients with SGLT2 inhibitors and 17.4% (2,445/14,067) with placebo, which showed a significantly higher risk associated with SGLT2 inhibitors (RR: 1.18, 95% CI: 1.10 to 1.26, *P* < 0.001). Conversely, SGLT2 inhibitors was associated with favorable effects on hypertension (RR: 0.61, 95% CI: 0.50 to 0.75, *P* < 0.001) and edema/peripheral edema (RR 0.49, 95% CI: 0.33 to 0.72, *P* < 0.001). No statistical difference was found in the incidence of other known AEs (musculoskeletal disorders, gastrointestinal disorders, metabolism, and nutrition).

#### Subgroup and Sensitivity Analyses

**Figure 2:**
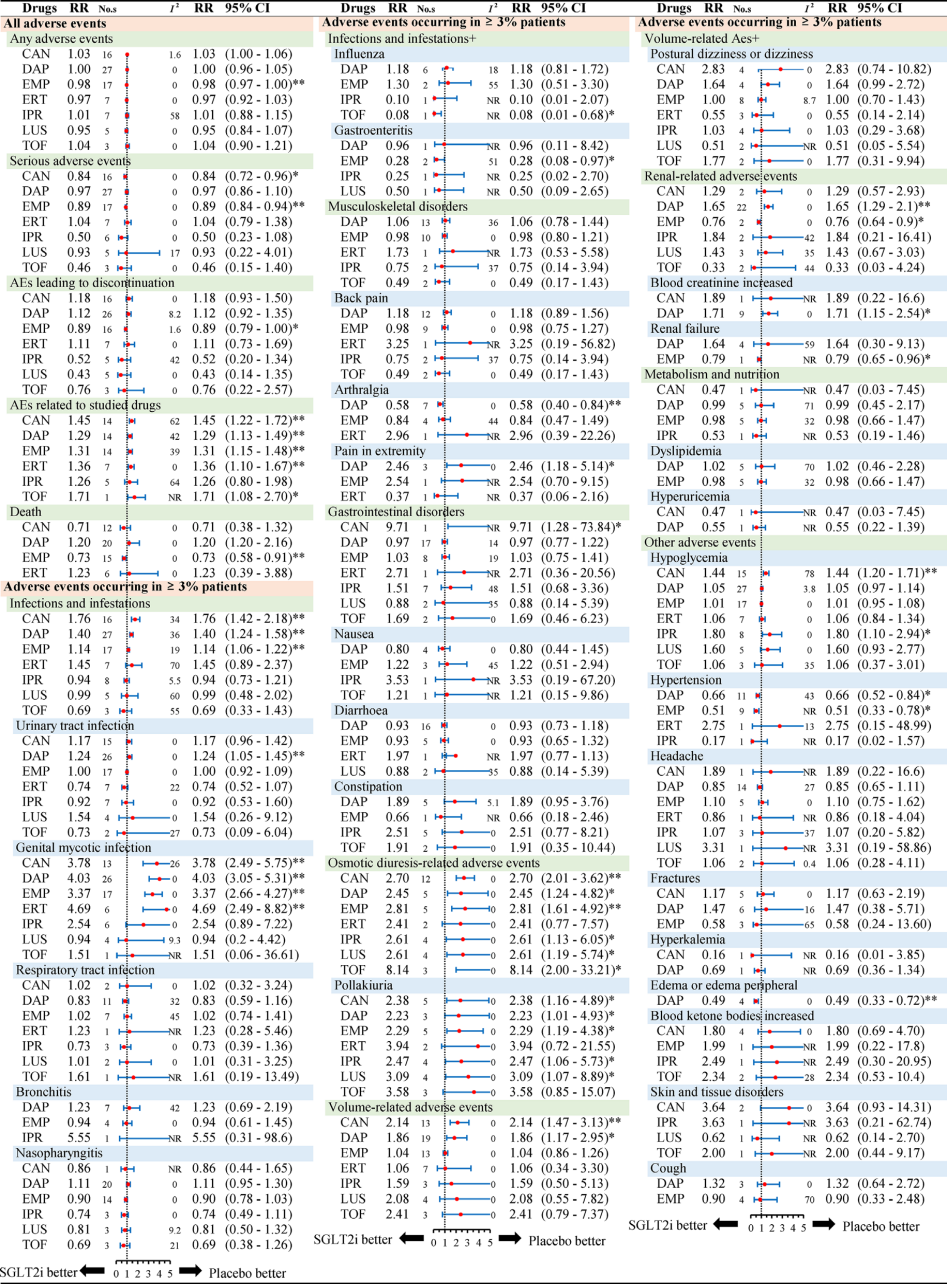
Subgroup analysis of relative risk of adverse events reported for each SGLT2i in comparison to placebo. Different colors represent different menu levels (pink > green > blue). Abbreviations: No.s, number of studies; NR, not required; CAN, canagliflozin; DAP, dapagliflozin; EMP, empagliflozin; IPR, ipragliflozin; TOF, tofogliflozin; LUS luseogliflozin; ERT ertugliflozin.* *P* < 0.05, ** *P* < 0.001.

**Figure 2** presents the subgroup results of individual SGLT2 inhibitors. The relationship between AEs and follow-up (less than 26 weeks or more than 26 weeks) or monotherapy or multi-therapy were listed in **Table S4**.

Considering overall safety by individual SGLT2 inhibitors, we noted a lower risk of serious adverse events in canagliflozin (RR 0.84, 95% CI: 0.72 to 0.96, *P* < 0.05) and empagliflozin (RR 0.89, 95% CI: 0.84 to 0.94, *P* < 0.001) when compared to placebo. For death, there was a significantly lower risk in empagliflozin (RR 0.73, 95% CI: 0.58 to 0.91, *P* < 0.001) than placebo (**Figure 2**). With regard to death by different follow-ups, SGLT2 inhibitors were associated with a lower risk of death in follow-up duration ≥26 weeks (RR 0.77, 95% CI: 0.63 to 0.94, *P* = 0.0011) (**Table S4**).

Considering the risk of AEs by different treatment, most results were consistent with the primacy analyses. As for infection and infestations, three SGLT2 inhibitors (canagliflozin, dapagliflozin, empagliflozin) were significantly associated with a higher risk than placebo. For UTI, only dapagliflozin was associated with a higher risk (RR 1.24, 95% CI: 1.05 to 1.45, *P* < 0.001). With regards to GMI, four SGLT2 inhibitors (canagliflozin, dapagliflozin, empagliflozin, and ertugliflozin) were significantly associated with a higher risk. Canagliflozin and dapagliflozin were different with respect to the volume-related AEs. As for renal-related AEs, dapagliflozin and empagliflozin showed a significant difference as compared to placebo. Dapagliflozin was significantly associated with a higher risk of blood creatinine increased effects (RR 1.71, 95% CI: 1.15 to 2.54, *P* < 0.05), while empagliflozin was associated with a lower risk of renal failure (RR 0.79, 95% CI: 0.65 to 0.96, *P* < 0.05). For skin and tissue disorders, all types of SGLT2 inhibitors were not different as compared to placebo. In addition, dapagliflozin and ipragliflozin were different in the context of hypoglycemia. Collectively, subgroup analyses by individual SGLT2 inhibitors showed some differences as above, but other analyses by length of follow-up or monotherapy or multitherapy were in line with the primary results (**Table S4**).

The results of sensitivity analysis, as shown in **Table S5**, were not altered after omitting each of the studies. In addition, we conducted further analyses by excluding 13 studies (13 researches contained two period of follow-up considered as 26 studies) with short follow-ups, and the results were consistent with primacy analyses. The full detailed results of the latter analyses were presented in **Table S6**.

#### Publication Bias

As shown in **Figure S2 (A–E)**, we did not observe potential publication bias by qualitative funnel plots and Begg’s test and Egger’s test.

## Discussion

This study was a meta-analysis to comprehensively evaluate the non-cardiovascular safety of SGLT2 inhibitors, including 78 publications (83 comparisons) with 7 individual SGLT2 inhibitors in patients with T2DM. SGLT2 inhibitors were associated with reduced risk of death and serious adverse effects when compared with placebo. However, SGLT2 inhibitors accompanied with a higher risk of infections, especially for GMI. Other significant AEs observed in SGLT2 inhibitors were osmotic diuresis-related AEs, volume-related AEs, renal-related AEs, increased blood ketone bodies, and skin/tissue disorders. These results were consistent across the key subgroups. The lower risk of all causes of death in T2DM patients treated with SGLT2 inhibitors may be associated with its remarkable cardiovascular benefits. Results from Empagliflozin Cardiovascular Outcome Event Trial in Type 2 Diabetes Mellitus Patients (EMPA-REG OUTCOME) indicated that T2DM patients accompanying with high cardiovascular risk achieved a lower risk of all causes of death (HR 0.68, 95% CI: 0.57 to 0.82) and serious adverse event (incidence rate 38.2% vs. 42.3%) with the treatment of empagliflozin than placebo (Zinman et al., 2015). In addition, canagliflozin cardiovascular assessment study (CANVAS) based on over 10,000 patients with T2DM reported a 13% reduction in the risk of all causes of death (HR 0.87, 95% CI: 0.74 to 1.01). Moreover, a 7% reduction of serious adverse effects (HR 0.93, 95% CI: 0.87 to 1.00) was observed for canagliflozin compared with placebo (Neal et al., 2017). Recently, two large observational studies also reported a remarkably lower rate (nearly 50% lower mortality rate) of all causes of death in all patients with T2DM who were treated with SGLT2 inhibitors, rather than those T2DM patients with a high cardiovascular risk (Nystrom et al., 2017; Kosiborod et al., 2018). However, these observational studies might be affected by time-related biases, which inevitably lead to the exaggeration on benefits observed with SGLT2 inhibitors. (Nystrom et al., 2017; Kosiborod et al., 2018; Suissa, 2018).

In this study, we selected all double-blinded, placebo-controlled RCTs to evaluate the overall adverse events of SGLT2 inhibitors compared with placebo. Our results confirmed that SGLT2 inhibitors were associated with a lower risk of death and serious adverse events when compared with placebo. Drug-specified subgroup analysis showed that empagliflozin lowers rates of all-cause mortality. As for serious adverse events, both empagliflozin and canagliflozin were associated with lower risks. In general, cardiovascular death was the main factor of all cause death. The lower risk of death in T2DM patients treated with SGLT2 inhibitors may result in its remarkable cardiovascular benefits (Zhang et al., 2018). However, it was still uncertain whether SGLT2 inhibitors were associated with a lower risk of death in T2DM patients with low risk of cardiovascular diseases.

Infection was a main non-cardiovascular AE for SGLT2 inhibitors. Our findings substantiate concern that SGLT2 inhibitors, mainly from dapagliflozin, canagliflozin, and empagliflozin, were associated with a significantly increased risk of infections. And, only dapagliflozin, but not other SGLT2 inhibitors, might confer a higher risk of UTI compared with placebo. These results were in accordance with previous meta-analyses (Liu et al., 2017; Puckrin et al., 2018). Similarly, the increased risk of GMI occurred across four types of SGLT2 inhibitors (dapagliflozin, canagliflozin, empagliflozin, and ertugliflozin). There was no increased risk of respiratory tract infection, bronchitis, and nasopharyngitis when administrated with SGLT2 inhibitors in patient with T2DM. Meanwhile, our results indicated that tofogliflozin was associated with a lower risk of influenza whereas empagliflozin was associated with a lower risk of gastroenteritis. However, there still needs more RCTs or real-world studies for more insight.

Several pathophysiologic mechanisms might explain the reason of high risks of infections, especially for GMI. SGLT-2 inhibitors enhanced urinary glucose excretion, leading to a proliferation of fungi and/or other microorganisms in the genitourinary tract, which might contribute to a higher risk of genital infections and/or poor clinical outcomes (Geerlings et al., 2014). Whereas the underlying mechanism for the increased risk of UTI with dapagliflozin but no other SGLT2 inhibitors was unclear. The unobserved UTI with empagliflozin and canagliflozin might be due to excluding patients with a history of genitourinary infections in these trials (Puckrin et al., 2018).

The osmotic diuresis was closely related to glycemic level and blood pressure. SGLT2 inhibitors lowered glycemic level in T2DM patients and reduce blood pressure, secondary to mild diuresis consequent to SGLT2 inhibitors-induced glucosuria. SGLT2 inhibitors induced diuresis might potentially contribute to AEs of volume reduction, leading to events such as hypotension and syncope (Johnsson et al., 2016). In contrast to an earlier pooled study (Johnsson et al., 2016), we identified a significant association between SGLT2 inhibitors and the risk of osmotic diuresis–related AEs and volume-related AEs. More specifically, SGLT2 inhibitors had also shown an increase in pollakiuria. On the other hand, SGLT2 inhibitors might be associated with a lower risk of hypertension and edema. Unlike other osmotic diuretic agents, such as mannitol, SGLT2 inhibitors were restricted to the renal tubules. Therefore, with less water reabsorption from the urine, SGLT2 inhibitors lead to the increased osmotic pressure in the renal tubules. Since the osmotic effects of SGLT2 inhibitors is targeted at the kidneys and result in some degree of vascular contraction, they might, at least in part, be beneficial in patients with CV complications such as heart failure (Zinman et al., 2015). There was potential concern that a raised incidence of AEs of volume reduction might increase the risk for falls and fractures. Although SGLT2 inhibitor therapy was associated with an increase in AEs of volume reduction, there was no increase in fractures in patients receiving SGLT2 inhibitors.

SGLT2 inhibitors were associated with a good control ability of glycemia, body weight, blood pressure, and serum urate levels. Possibly as a consequence of these pleiotropic effects, clinical trials conducted in T2DM patients with high cardiovascular risk have demonstrated that SGLT2 inhibitors can reduce not only relevant cardiovascular risk but also renal adverse outcomes (Zinman et al., 2015; Neal et al., 2017; Thomas and Cherney, 2018; Zhao et al., 2018). Not totally consistent with a previous meta-analysis, SGLT2 inhibitors were significantly associated with a greater risk of composite renal events than placebo (Tang et al., 2017). Individual SGLT2 inhibitor with adverse renal outcomes was showed as follows: dapagliflozin was significantly associated with a great risk of renal-related AEs, especially for elevating blood creatinine, and empagliflozin seemed to confer a lower renal-related AE risk, especially for renal failure. As for the other five SGLT2 inhibitors, this meta-analysis did not show association with risks of renal adverse AEs. Large clinical trials had been conducted for SGLT2 inhibitors as per of post-marketing and drug efficacy and safety studies for new antidiabetic drugs. So far, two SGLT2 inhibitors have been evaluated: empagliflozin in the EMPA-REG OUTCOME trial (Zinman et al., 2015) and canagliflozin in CANVAS Program (Neal et al., 2017). Results from the study of empagliflozin showed that it can improve renal outcomes defined by reduced risk of incidents or worsening nephropathy, decreased progression to macroalbuminuria, lowered incidence of renal-replacement therapies, and occurrence of doubling of serum creatinine (Zinman et al., 2015). Recently, the CANVAS Program reported similar renal protective effects of canagliflozin in T2DM patients (Neal et al., 2017). Canagliflozin reduced the occurrence of progression to albuminuria and increased the occurrence of regression of albuminuria. Consistent with EMPA-REG OUTCOME trial, our finding showed empagliflozin had protection effect of kidney. Whereas different from the CANVAS Program, our findings showed that canagliflozin was not associated with risks of renal-related AEs. Our study defined the composite renal outcomes as renal-related AEs, blood creatinine increase or eGFR decrease, blood urea increase, renal impairment, renal failure, urine β2 macroglobulin increase, and microalbuminuria. Thus, differential definitions of what a renal outcome might be the consequence of these different effects. SGLT2 inhibitors and their effects on renal outcome in T2DM had multiple effects, meaning that SGLT2 inhibitors could induce an early, reversible reduction in GFR and preserve GFR in the long-term in T2DM (Nespoux and Vallon, 2018).

SGLT2 inhibitors were associated with a higher risk of hypoglycemia as compared with placebo. However, this phenomenon was not observed when SGLT2 inhibitors were administrated as monotherapy.

There remain controversies on the risk of increased blood ketone bodies caused by SGLT2 inhibitors. The Food and Drug Administration (FDA) had issued warnings about occurrences of ketoacidosis during post-marketing studies of SGLT2 inhibitors (FDA, 2015b), while some early studies did not support this finding (Erondu et al., 2015; Peters et al., 2015; Monami et al., 2017). In our meta-analysis, the results showed SGLT2 inhibitors were significantly associated with a higher risk of increased blood ketone bodies as compared with placebo.

The major strength of this study was the comprehensive and exquisite evaluation of the non-cardiovascular safety of SGLT2 inhibitors. Compared with previous study (Radholm et al., 2018), our meta-analysis has several advantages: (1) our research question was more specific to non-cardiovascular adverse events outcomes; (2) this was the first meta-analysis to assess the comparative safety of SGLT2 inhibitors on all non-cardiovascular adverse events; (3) AEs occurring in ≥3% patients were systematically identified; and (4) subgroup analyses about individual SGLT2 inhibitors, different follow-ups, and monotherapy or multitherapy were conducted.

Certainly, there were several limitations in this study. Firstly, we only included double-blinded, placebo-controlled RCTs and did not evaluate low incidence (<3% patients) AEs. Secondly, the majority of the trials (especially those on canagliflozin and empagliflozin) were less likely to report specific adverse outcomes in their full publications, but additional data were obtained from the U.S. National Library of Medicine web (*ClinicalTrials.gov*) to minimize the risk of reporting bias. Thirdly, the variation in background treatments and patient characteristics across RCTs might contribute to the heterogeneity, although we found low statistical heterogeneity in this meta-analysis. Fourthly, 5 of the 78 publications which were included in this study had two different follow-up durations, and the safety results over each distinct follow-up period were considered as 2 separated studies. In addition, 8 researches also had two different follow-up durations, but these results were reported in 16 publications. Some patient cohorts were included twice in this study. This might introduce bias in this study. Fifthly, we did not assess the correlation between drug dosage and AEs in this study as we listed in the subgroup analysis in our previous protocol. There were 36 doses of 7 SGLT2 inhibitors and 40 safety outcome indicators in this study. Because of the excessive number of outcomes and doses, we did not assess it in this article. Finally, overall adverse events for SGLT2 inhibitors—the exception being for empagliflozin, dapagliflozin and canagliflozin—remain uncertain because of the lack of sufficient data.

## Conclusion

Our meta-analysis showed that treatment of T2DM patients with SGLT2 inhibitors is associated with significant lower risk of serious adverse events and death. The main findings in the present study were that SGLT2 inhibitors were associated with a higher risk of osmotic diuresis–related AEs and volume-related AEs, which was not reported in other meta-analysis. Furthermore, we found SGLT2 inhibitors were associated with a higher risk of increase in blood ketone bodies and hypoglycemia when pooling the monotherapy and multitherapy. Consistent with other previous meta-analysis, we found that the main associated risk of SGLT2 inhibitors were infections especially for GMI. Likewise, SGLT2 inhibitors were associated with a higher risk of renal-related AEs. These results called for the strict monitoring of SGLT2 inhibitors-specific AEs in clinic.

## Data Availability

All datasets analyzed for this study are included in the manuscript and/or the **Supplementary files**.

## Author Contributions

Conceptualization: F-HS, HL, Z-CG, and H-WL. Data curation: F-HS, HL, LS, Y-HJ, Y-MH, and X-YL. Formal analysis: F-HS, HL, and Z-CG. Funding acquisition: F-HS, HL, Z-CG, and H-WL. Methodology: F-HS, HL, and Z-CG. Writing – original draft: F-HS and HL. Language polishing: ZZ. Writing – review and editing: Z-CG, JM and H-WL.

## Funding

This article is supported by “the Clinical Pharmacy Innovation Research Institute of Shanghai Jiao Tong University School of Medicine (2019)” (CXYJY2019QN004 and CXYJY2019ZD001), “the Fundamental Research Funds for the Central Universities” (No. 17JCYB11), “the pharmaceutical fund of college of medicine, Shanghai Jiaotong University” (No. JDYX2017QN003), “the fund of Shanghai Pharmaceutical Association” (No. 2015-YY-01-20), and “Program for Key but Weak Discipline of Shanghai Municipal Commission of Health and Family Planning” (2016ZB0304).Shanghai Municipal Education Commission—Gaofeng Clinical Medicine (20181807) and National Natural Science Foundation of China (81670728).

## Conflict of Interest Statement

The authors declare that the research was conducted in the absence of any commercial or financial relationships that could be construed as a potential conflict of interest.
