# Supplementary material for: Appraisal of Non-Cardiovascular Safety for Sodium–Glucose Co-Transporter 2 Inhibitors: A Systematic Review and Meta-Analysis of Placebo-Controlled Randomized Clinical Trials
Source: Front Pharmacol. 2019 Sep 19;10:1066. doi: 10.3389/fphar.2019.01066 (PMC6764217; doi:10.3389/fphar.2019.01066)
Supplement: Supplementary file 1 [file Table_1.pdf]

SUPPLEMENTAL FILE

Appraisal of non-cardiovascular safety for sodium-glucose co-transporter 2 inhibitors: A systematic review and meta-analysis of placebo-controlled randomized clinical trials

Tables

Supplementary Table 1. Search strategy used in Sep 5th, 2018 3

Supplementary Table 2. Treatment and baseline characteristics of included studies and treatment arms........ 6

Supplementary Table 3. Quality assessment results of included randomized controlled trials 8

Supplementary Table 4. Subgroup analysis of main safety outcomes of SGLT2 inhibitors versus Placebo........ 12

Supplementary Table 5. Sensitivity analysis for main safety outcomes........ 21

Supplementary Table 6. Further researches of relative risks of adverse events reported for SGLT2 inhibitors in comparison to placebo in 70 RCTs……………………. ……………………. ……………………. ……………………. ……………………. ……………………. ………………32

Figures

Supplementary Figure 1. Flow diagram for the selection of eligible RCTs. 34

Supplementary Figure 2. Funnel plot of main safety indexes........ 35

References……………………………………………………36

Abbreviations in Supplementary Figures

RCT: Randomized controlled trial; SGLT2 inhibitors: sodium-glucose co-transporter 2 inhibitors.

Supplementary Table 1. Search strategy used in Sep 5th, 2018

Literature databases	Search items	Items found
PUBMED	“Sodium glucose co-transporter”[Title/Abstract] OR “SGLT2”[Title/Abstract] OR“SGLT-2”[Title/Abstract]OR “SGLT 2”[Title/Abstract] OR “Tofogliflozin”[MeSH Terms] OR “Tofogliflozin”[Title/Abstract] OR “Apleway”[Title/Abstract] OR “Deberza”[Title/Abstract] OR“CSG452”[Title/Abstract]OR “Empagliflozin” [MeSH Terms] OR “Empagliflozin” [Title/Abstract] OR“Jardiance”[Title/Abstract] OR “dapagliflozin”[MeSH Terms] OR “dapagliflozin”[Title/Abstract] OR “Farxiga”[Title/Abstract]) OR “Forxiga”[Title/Abstract])OR “BMS-512148”[Title/Abstract]) OR “Canagliflozin”[MeSH Terms] OR “Canagliflozin”[Title/Abstract] OR “Invokana”[Title/Abstract]) OR “Sotagliflozin”[MeSH Terms] OR “Sotagliflozin”[Title/Abstract] OR “LX4211”[Title/Abstract]) “luseogliflozin”[MeSH Terms] OR “luseogliflozin”[Title/Abstract] OR “Lusefi”[Title/Abstract]) “ipragliflozin”[MeSH Terms] OR “ipragliflozin”[Title/Abstract] OR “Suglat”[Title/Abstract])OR “remogliflozin”[MeSH Terms] OR “remogliflozin”[Title/Abstract] OR “BHV091009”[Title/Abstract]) OR “sergliflozin”[MeSH Terms] OR “sergliflozin”[Title/Abstract] OR “GW869682X”[Title/Abstract])OR “ertugliflozin”[MeSH Terms] OR “ertugliflozin”[Title/Abstract] OR “MK-8835”[Title/Abstract])OR “PF-04971729”[Title/Abstract] AND “Randomized Controlled Trial”[Publication Type] OR “RCTs” [Title/Abstract]) OR “RCT” [Title/Abstract])	3407
EMBASE	‘Tofogliflozin’/exp OR ‘Tofogliflozin’:ti,ab,kw OR	4364

	‘Apleway’:ti,ab,kw OR ‘Deberza’:ti,ab,kw OR ‘CSG452’:ti,ab,kw OR ‘Empagliflozin’/exp OR ‘Empagliflozin’:ti,ab,kw OR ‘Jardiance’:ti,ab,kw OR ‘dapagliflozin’/exp OR ‘dapagliflozin’:ti,ab,kw OR ‘Farxiga’:ti,ab,kw OR ‘Forxiga’:ti,ab,kw OR ‘BMS-512148’:ti,ab,kw OR ‘Canagliflozin’/exp OR ‘Canagliflozin’:ti,ab,kw OR ‘Invokana’:ti,ab,kw OR ‘Sotagliflozin’/exp OR ‘Sotagliflozin’:ti,ab,kw OR ‘LX4211’:ti,ab,kw OR ‘luseogliflozin’/exp OR ‘luseogliflozin’:ti,ab,kw OR ‘Lusefi’:ti,ab,kw OR ‘ipragliflozin’/exp OR ‘ipragliflozin’:ti,ab,kw OR ‘Suglat’:ti,ab,kw OR ‘remogliflozin’/exp OR ‘remogliflozin’:ti,ab,kw OR ‘BHV091009’:ti,ab,kw OR ‘sergliflozin’/exp OR ‘sergliflozin’:ti,ab,kw OR ‘GW869682X’:ti,ab,kw OR ‘ertugliflozin’/exp OR ‘ertugliflozin’:ti,ab,kw OR ‘MK-8835’:ti,ab,kw OR ‘PF-04971729’:ti,ab,kw OR ‘Sodium glucose co-transporter’:ti,ab,kw OR ‘SGLT2’:ti,ab,kw OR ‘SGLT-2’:ti,ab,kw OR ‘SGLT 2’:ti,ab,kw	
COCHRANE	MeSH descriptor: [Tofogliflozin] OR Tofogliflozin: ti,ab,kw OR Apleway: ti,ab,kw OR Deberza: ti,ab,kw OR CSG452: ti,ab,kw OR MeSH descriptor: [Empagliflozin] OR Empagliflozin: ti,ab,kw OR Jardiance: ti,ab,kw OR MeSH descriptor: [dapagliflozin] OR dapagliflozin: ti,ab,kw OR Farxiga: ti,ab,kw OR Forxiga: ti,ab,kw OR BMS-512148: ti,ab,kw OR MeSH descriptor: [Canagliflozin] OR Canagliflozin: ti,ab,kw OR Invokana: ti,ab,kw OR MeSH descriptor: [Sotagliflozin] OR Sotagliflozin: ti,ab,kw OR LX4211: ti,ab,kw OR MeSH descriptor: [luseogliflozin] OR luseogliflozin: ti,ab,kw OR Lusefi: ti,ab,kw OR MeSH descriptor: [ipragliflozin] OR ipragliflozin: ti,ab,kw OR Suglat: ti,ab,kw OR MeSH descriptor: [remogliflozin] OR remogliflozin: ti,ab,kw	1641

	OR BHV091009: ti,ab,kw OR MeSH descriptor: [sergliflozin] OR sergliflozin: ti,ab,kw OR GW869682X: ti,ab,kw OR MeSH descriptor: [ertugliflozin] OR ertugliflozin: ti,ab,kw OR MK-8835: ti,ab,kw OR PF-04971729: ti,ab,kw OR Sodium glucose co-transporter: ti,ab,kw OR SGLT2: ti,ab,kw OR SGLT-2: ti,ab,kw OR SGLT 2: ti,ab,kw	
Overall		9412
Duplication		3847

Supplementary TABLE 2. Treatment and baseline characteristics of included studies and treatment arms.

	Author (year)	NCT number	Treatments	Background therapy	Sample size (n)	HbA1c (%)	BMI (kg/m2)	Age (year)	Sex (male%)	DM duration	Follow -ups
1	Bode,B 2013[1]	NCT1106651	CANA 100mg,300mg	AHA	714	7.7	31.6	63.6	55.5	11.7	26
2	Bode,B 2015[2]	NCT01106651	CANA 100mg,300mg	AHA	714	7.7	31.6	63.6	55.5	11.7	104
3	Inagaki,N 2013[3]	NCT01022112	CANA50mg;100mg;200mg;300mg	DE	382	8.1	25.7	57.4	68.1	NR	12
4	Inagaki,N 2014[4]	NCT01413204	CANA100mg,200mg	DE	272	8	25.6	58	70.5	5.4	24
5	Inagaki N 2016[5]	NCT02220920	CANA 100mg	INS±DE	146	8.9	26.5	58	63.7	13.8	16
6	Ji, L 2015[6]	NCT01381900	CANA 100mg,300mg	MET±SU	676	8	25.7	56.2	53.6	6.7	18
7	Kadowaki,T 2017[7]	NCT02354235	CANA100mg	TENE	138	8	26	57.2	77.5	7.4	24
8	Neal, B 2015[8]	NCT01032629	CANA 100mg,300mg	INS	2072	8.3	33.1	62.7	66	16.2	52
9	Qiu, R 2014[9]	NCT01340664	CANA 100mg,300mg	MET	279	7.6	32.5	57.4	46.6	7	18
10	Rodbard, H.W 2016[10]	NCT01989754	CANA 100mg to 300mg	MET+SIT	213	8.5	32	57.4	56.8	9.9	26
11	Stenlof, K 2013[11]	NCT01081834	CANA 100mg,300mg	DE+AHA	584	8	31.6	55.4	44.2	4.3	26
12	Wilding, JP 2013[12]	NCT01106625	CANA 100mg,300mg	MET+SU	469	8.1	33.1	56.8	51	9.6	26,52
13	Yale, JF 2013[13]	NCT01064414	CANA 100mg,300mg	AHA	269	8	33	68.5	60.6	16.3	26
14	Yale, JF 2014[14]	NCT01064414	CANA 100mg,300mg	AHA	269	8	33	68.5	60.6	16.3	52
15	Yale JF 2017[15]	NCT01032629	CANA 100mg,300mg	SU	215	8.2	25.7	65.1	54.1	9.2	52
16	Araki, E 2016[16]	NCT02157298	DAPA5mg	INS+DPP4i or not	183	8.3	26.6	58	70.9	15	16
17	Bailey, CJ 2012[17]	NR	DAPA1mg,2.5mg,5mg	drug-naive	282	7.9	31.8	53	50.1	1.4	24
18	Bailey, CJ 2013[18]	NCT00528879	DAPA2.5mg,5mg,10mg	MET	546	8.1	NR	NR	NR	NR	102
19	Bailey CJ 2015[19]	NCT 00528372	DAPA2.5mg,5mg,10mg	DE	274	7.9	NR	52.2	48.2	1.9	102
20	Bolinder, J 2012[20]	NCT00855166	DAPA 10mg	MET	182	7.2	31.9	60.7	55.6	5.8	24
21	Bolinder, J 2014[21]	NCT00855166	DAPA 10mg	MET	182	7.2	31.9	60.7	55.6	5.8	102
22	Cefalu, WT 2015[22]	NCT01042977	DAPA10mg	AHA exp ROS	922	8.1	32.8	62.9	69.3	12.4	52
23	Jabbour, SA 2014[23]	NCT00984867	DAPA10mg	SIT±MET	451	7.9	NR	54.9	54.8	5.7	24,48
24	Ji, L 2014[24]	NCT01095653	DAPA 5mg,10mg	NR	393	8.3	25.6	51.3	65.4	0.3	24
25	Kaku, K 2013[25]	NCT00972244	DAPA1.0mg,2.5mg.5mg,10mg	DE	279	8.1	NR	55	77	4.83	12
26	Kaku, K 2014[26]	NR	DAPA 5mg,10mg	DE	261	7.5	25.4	58.8	59.4	4.9	24
27	Kohan, DE 2014[27]	NCT00663260	DAPA 5mg,10mg	DE±AHA	252	8.3	NR	67	65.1	16.9	104
28	Leiter, LA 2014[28]	NCT01042977	DAPA 10mg	INS	965	8.1	32.9	63.8	66.9	13.3	52
29	List, JF 2009[29]	NCT00263276	DAPA2.5mg,5mg,10mg,20mg, 50mg	NR	333	7.8	31.8	54.2	50.7	NR	12
30	Mathieu, C 2015[30]	NCT01646320	DAPA10mg	SAXA+MET	320	8.2	31.7	55.1	45.6	7.6	24

31	Mathieu, C 2016[31]	NCT01646320	DAPA10mg	SAXA+MET	320	8.2	31.7	55.1	45.6	7.6	52
32	Matthaei, S 2015[32]	NCT01392677	DAPA10mg	MET+SU	218	8.2	NR	NR	48.6	NR	24,52
33	Rosenstock, J 2012[33]	NCT00683878	DAPA 5mg,10mg	PIO+MET orSUorTZD	420	8.4	NR	53.5	49.5	5.5	24
34	Schumm-Draeger, P M.2015[34]	NCT01217892	DAPA2.5mg bid,5mg bid and 10mg qd	MET	400	7.8	32.6	57.7	44.9	16	
35	Stroje K 2011[35]	NCT00680745	DAPA2.5mg,5mg,10mg	GLI	596	8.1	NR	59.8	48.1	7.4	24
36	Strojek K 2014[36]	NCT00680745	DAPA2.5mg,5mg,10mg	GLI	596	8.1	NR	59.8	48.1	7.4	48
37	Weber, MA 2016[37]	NCT01195662	DAPA10mg	AHA	449	8	NR	56.5	55	7.5	12
38	Wilding, JP 2012[38]	NCT00673231	DAPA 2.5mg,5mg,10mg	INS+OAD or not	807	8.5	33.1	59.3	47.7	13.6	24
39	Wilding, JP 2014[39]	NCT00673231	DAPA 2.5mg,5mg,10mg	INS+OAD or not	807	8.5	33.1	59.3	47.7	13.6	104
40	Yang W 2016[40]	NCT01095666	DAPA 5mg,10mg	MET	444	8.1	26.1	53.7	54.3	4.9	24
41	Barnett, AH 2014[41]	NCT01164501	EMPA10mg,25mg	AHA	738	8	30.7	63.9	58.3	NR	24
42	Ferrannini, E 2013[42]	NCT00789035	EMPA 5mg,10mg,25mg	no OAD $	326	7.9	28.5	58	52	NR	12
43	Haering, HU 2015[43]	NCT01289990	EMPA10mg,25mg	MET+SU	666	8.1	28.2	57.1	50.9	NR	72
44	Haring, HU 2013[44]	NCT01159600	EMPA10mg,25mg	MET+SU	666	8.1	28.2	57.1	51	NR	24
45	Haring, HU 2014[45]	NCT01159600	EMPA10mg,25mg	MET	637	7.9	29.2	55.7	57	NR	24
46	Kadowaki,T 2014[46]	NCT01193218	EMPA5mg,10mg,25mg,50mg	DE	547	8	25.5	57.5	75	NR	12
47	Kovacs, CS 2014[47]	NCT01210001	EMPA10mg,25mg	PIO±MET	498	8.1	29.2	54.5	48.4	NR	24
48	Merker, L 2015[48]	NCT01159600, NCT01289990	EMPA10mg,25mg	DE+MET	637	7.9	29.2	55.7	56.7	NR	72
49	Roden, M 2013[49]	NCT01177813	EMPA10mg,25mg	No	676	7.9	28.4	55	61	NR	24
50	Roden, M 2015[50]	NCT01289990	EMPA10mg,25mg	DE	676	7.9	28.4	55	61.3	NR	72
51	Rosenstock, J 2013[51]	NCT00749190	EMPA1mg,5mg,10mg,25mg,50 mg	MET±OAD	424	7.9	31.4	58.5	50.3	NR	12
52	Rosenstock, J 2014[52]	NCT01306214	EMPA10mg,25mg	AHA	563	8.3	34.8	56.7	45	NR	52
53	Rosenstock, J 2015[53]	NCT01011868	EMPA10mg,25mg	basal insulin	494	8.2	32.2	58.8	56	NR	78
54	Ross, S 2015[54]	EU 2012-000905-53	EMPA 5mg bid, 10mg,12,5mg bid,25mg	MET	983	7.8	31.8	58.2	53.9	NR	16
55	Softeland, E 2017[55]	NCT01734785	EMPA10mg,25mg	MET and LINA	332	8	30.2	55.2	60.3	NR	24
56	Tikkanen, I 2016[56]	NCT01370005	EMPA10mg,25mg	AHA	823	7.9	32.6	60.2	60.1	NR	12
57	Zinman, B 2016[57]	NCT01131676	EMPA10mg,25mg	AHA	7020	8.1	30.6	63.1	71.5	NR	161
58	Fonseca, VA 2013[58]	NCT01071850	IPRA 12.5, 50,150,300mg	DE±AHA	342	7.9	31.1	53.7	50	4.6	12
59	Ishihara, H 2016[59]	NCT02175784	IPRA50mg	INS±DPP4i	262	8.7	25.9	58.9	61.2	3	16
60	Kashiwagi, A 2014[60]	NCT00621868	IPRA 12.5, 25, 50,100mg	no AHA	361	8.3	25.7	55.9	64.7	1.5	12
61	Kashiwagi, A 2015A[61]	NCT01135433	IPRA50mg	MET	168	8.3	25.8	56.7	58.9	1.8	24
62	Kashiwagi, A 2015B[62]	NCT01316094	IPRA50mg	DE+AHA	165	7.5	25.6	64.4	78.1	2.2	24

63	Kashiwagi, A 2015C[63]	NCT01057628	IPRA50mg	noAHA	129	8.3	25.4	59.4	69.7	1.5	16
64	Lu, CH 2016[64]	NCT01505426	IPRA50mg	MET	171	7.7	26.8	53.7	45.3	1.4	24
65	Wilding, JP 2013[65]	NCT01117584	IPRA 12.5, 50,150,300mg	MET	342	7.8	31.7	57.4	51.2	5.9	12
66	Ikeda, S 2015[66]	NCT00800176	TOFO2.5mg,5mg,10mg,20mg,40mg	DE ±MET	394	8	30.5	55.1	53	5.6	12
67	Kaku, K 2014[67]	Japic CTI-101349	TOFO10mg,20,40mg	DE	230	8.4	25.5	57.2	66.8	6.4	24
68	Terauchi, Y 2017[68]	NCT02201004	TOFO20mg	INS±DPP4i	210	8.5	26.2	58.2	65.4	14.1	16
69	SeinoY 2014A[69]	Japic CTI-101191	LUSE 1,2.5,5,10mg	noAHA	282	7.9	24.8	58	70.4	5	12
70	SeinoY 2014B[70]	JapicCTI-090908	LUSE 0.5,2.5,5mg	noAHA	236	8.1	25	57	67.8	6	12
71	SeinoY 2014C[71]	JapicCTI-111661	LUSE2.5mg	DE	158	8.2	25.7	59.3	73.4	6.3	24
72	SeinoY 2015[72]	JapicCTI-111507	LUSE2.5mg	SU	221	8.1	24.7	60.8	72.4	7.6	24
73	SeinoY 2018[73]	JapicCTI-142582	LUSE2.5mg	INS	233	8.7	25.3	57.3	69.9	11.8	16
74	Amin NB 2015[74]	NCT01059825	ERTU1,5,10,25mg	MET±OAD	273	8.1	30.4	54.7	63.4	6.3	12
75	Terra, SG 2017[75]	NCT01958671	ERTU5mg,15mg	DE	461	8.2	33	56.4	56.6	5	26
76	Dagogo-JackS 2018[76]	NCT02036515	ERTU5mg,15mg	MET+SIT	462	8	30.8	59.1	56.9	9.5	26,52
77	Grunberger G 2018[77]	NCT01986855	ERTU5mg,15mg	AHA	467	8.2	32.5	67.3	49.5	14.2	26,52
78	Rosenstock, J 2018[78]	NCT02033889	ERTU5mg,15mg	MET	621	8.1	30.9	56.6	46.4	8	26

Abbreviations:

CANA: Canagliflozin; DAPA: Dapagliflozin; EMPA: Empagliflozin; IPRA: Ipragliflozin; TOFO: Tofogliflozin; LUSE: Luseogloflozin; ERTU: Ertuliflozin; AHA: Anti-hyperglycaemic agents; OAD: Oral antidiabetic drugs ; DE:Diet and exercises; MET: Metformin; INS: Insulin; SU: Sulfonylureas; TENE: Teneligliptin; SIT: Sitagliptin; DPP4i:Dipeptidyl peptidase-4 inhibitors; ROS: Rosiglitazone; PIO:Pioglitazone;TZD:Thiazolidinedione;SAXA:Saxagliptin;LINA:Linagliptin;NR: not reported.

Supplementary TABLE S3 Quality assessment results of included randomized controlled trials.

Study	Random sequence generation	Allocation concealment	Blinding of participants and personnel	Blinding of outcome assessment	Incomplete outcome data	Selective reporting	Other bias
**CANA**							
Bode, B 2013	L	L	L	L	L	L	L
Bode, B 2015	L	L	L	L	L	L	L
Inagaki, N 2013	L	L	L	L	L	L	L
Inagaki, N 2014	L	L	L	L	L	L	L
Inagaki N 2016	L	L	L	L	L	L	L
Ji, L 2015	L	L	L	L	L	L	L
Kadowaki, T 2017	L	L	L	L	L	L	L
Neal, B 2015	L	L	L	L	L	L	L
Qiu, R 2014	L	L	L	L	L	L	L
Rodbard, H.W 2016	L	L	L	L	L	L	L
Stenlof, K 2013	L	H	L	L	L	L	L
Wilding, JP 2013	L	L	L	L	L	L	L
Yale, JF 2013	L	L	L	L	L	L	L
Yale, JF 2014	L	L	L	L	L	L	L
Yale JF 2017	L	L	L	L	L	L	L
**DAPA**							
Araki, E 2016	L	L	L	L	L	L	L
Bailey, C.J 2012	L	L	L	L	L	L	L
Bailey, CJ 2013	L	L	L	L	L	L	L
Bailey CJ 2015	L	L	L	L	L	L	L

Bolinder, J 2012	L	L	L	L	L	L	L
Bolinder, J 2014	L	L	L	L	L	L	L
Cefalu, WT 2015	L	L	L	L	L	L	L
Jabbour, SA 2014	L	H	H	L	L	L	L
Ji, L 2014	L	L	L	L	L	L	L
Kaku, K 2013	L	L	H	L	L	L	L
Kaku, K 2014	L	H	H	L	L	L	L
Kohan, DE 2014	L	H	H	L	L	L	L
Leiter, LA 2014	L	H	H	L	L	L	L
List, JF 2009	L	H	H	L	L	L	L
Mathieu, C 2015	L	L	L	L	L	L	L
Mathieu, C 2016	L	L	L	L	L	L	L
Matthaei, S 2015	L	H	L	L	L	L	L
Rosenstock, J 2012	L	H	H	L	L	L	L
Schumm-Draeger, P. M.2015	L	L	L	L	L	L	L
Stroje K 2011	L	L	L	L	L	L	L
Strojek K 2014	L	L	L	L	L	L	L
Weber, MA 2016	L	L	L	L	L	L	L
Wilding, JP 2012	L	L	L	L	L	L	L
Wilding, JP 2014	L	L	L	L	L	L	L
Yang W 2016	L	H	H	L	L	L	L
**EMPA**							
Barnett, AH 2014	L	L	L	L	L	L	L
Ferrannini, E 2013	L	L	L	L	L	L	L
Haering, HU 2015	L	L	L	L	L	L	L

Haring, HU 2013	L	L	L	L	L	L	L
Haring, HU 2014	L	L	L	L	L	L	L
Kadowaki, T 2014	L	L	L	L	L	L	L
Kovacs, CS 2014	L	L	L	L	L	L	L
Merker, L 2015	L	L	L	L	L	L	L
Roden, M 2013	L	L	L	L	L	L	L
Roden, M 2015	L	L	L	L	L	L	L
Rosenstock, J 2013	L	L	L	L	L	L	L
Rosenstock, J 2014	L	L	L	L	L	L	L
Rosenstock, J 2015	L	L	L	L	L	L	L
Ross, S 2015	L	L	L	L	L	L	L
Softeland, E 2017	L	L	L	L	L	L	L
Tikkanen, I 2016	L	L	L	L	L	L	L
Zinman, B 2016	L	L	L	L	L	L	L
IPRA							
Fonseca, VA 2013	L	H	H	L	L	L	L
Ishihara, H 2016	L	L	L	L	L	L	L
Kashiwagi, A 2014	L	H	H	L	L	L	L
Kashiwagi, A 2015A	L	H	H	L	L	L	L
Kashiwagi, A 2015B	L	H	H	L	L	L	L
Kashiwagi, A 2015C	L	H	H	L	L	L	L
Lu, CH 2016	L	L	L	L	L	L	L
Wilding, JP 2013	L	H	H	L	L	L	L
TOFO							
Ikeda, S 2015	L	H	H	L	L	L	L

Kaku, K 2014	L	L	L	L	L	L	L
Terauchi, Y 2017	L	L	L	L	L	L	L
LUSE							
SeinoY 2014A	L	L	L	L	L	L	L
SeinoY 2014B	L	L	L	L	L	L	L
SeinoY 2014C	L	L	L	L	L	L	L
SeinoY 2015	L	L	L	L	L	L	L
SeinoY 2018	L	L	L	L	L	L	L
ERTU							
Amin NB 2015	L	L	L	L	L	L	L
Terra, SG 2017	L	L	L	L	L	L	L
Dagogo-Jack, S 2018	L	L	L	L	L	L	L
Grunberger, G 2018	L	L	L	L	L	L	L
Rosenstock, J 2018	L	L	L	L	L	L	L

Abbreviations: L, low risk of bias; H, high risk of bias.

Supplementary TABLE S4 Subgroup analysis of main safety outcomes of SGLT2 inhibitors versus Placebo

	No. of studies	I^2 (P value)	RR	95%CI	P
**Different Follow Ups**					
**All adverse events**					
**Any adverse events**					
Follow ups ≥26 w	32	0(0.504)	0.99	0.98 to 1.00	0.32
Follow ups <26 w	50	0(0.520)	1	0.97 to 1.02	0.875
**Serious adverse events**					
Follow ups ≥26 w	32	0(0.995)	0.9	0.86 to 0.95	<0.001
Follow ups <26 w	49	0(0.503)	0.82	0.69 to 0.97	0.02
**AEs leading to discontinuation**					
Follow ups ≥26 w	32	10.5(0.299)	1.03	0.91 to 1.15	0.661
Follow ups <26 w	46	0(0.473)	0.94	0.76 to 1.15	0.55
**AEs related to studied drugs**					
Follow ups ≥26 w	28	43.0(0.009)	1.35	1.23 to 1.48	<0.001
Follow ups <26 w	27	46.6(0.005)	1.32	1.15 to 1.51	<0.001
**Death**					
Follow ups ≥26 w	31	0(0.966)	0.77	0.63 to0.94	0.011
Follow ups <26 w	25	0(0.928)	0.83	0.33 to 2.09	0.696
**Adverse events occurring in ≥3% patients or other clinical interest**					
**Infections and infestations**					
Follow ups ≥26 w	32	71.1(<0.001)	1.48	1.31 to 1.66	<0.001
Follow ups <26 w	51	31.6(0.019)	1.13	1.20 to 1.24	0.012
**Urinary tract infection(UTI)**					
Follow ups ≥26 w	32	13.1(0.258)	1.09	0.97 to 1.20	0.126
Follow ups <26 w	46	0(0.995)	1.02	0.89 to 1.17	0.735
**Genital mycotic infection(GMI)**					
Follow ups ≥26 w	32	0(0.584)	4.26	3.55 to 5.09	<0.001

Follow ups <26 w	41	0(0.667)	2.72	2.07 to 3.56	<0.001
**Respiratory tract infection***					
Follow ups ≥26 w	9	30.8(0.172)	0.9	0.67 to 1.19	0.459
Follow ups <26 w	18	9.1(0.346)	0.94	0.73 to 1.20	0.641
**Bronchitis**					
Follow ups ≥26 w	8	3.8(0.401)	0.92	0.67 to 1.23	0.564
Follow ups <26 w	4	0(0.974)	3.91	1.18 to 12.87	0.025
**Nasopharyngitis**					
Follow ups ≥26 w	13	0(0.564)	1.05	0.90 to 1.21	0.491
Follow ups <26 w	31	0(0.917)	0.88	0.77 to 0.99	0.038
**Influenza**					
Follow ups ≥26 w	6	9.7(0.354)	1.32	0.93 to 1.87	0.114
Follow ups <26 w	4	49.8(0.113)	0.45	0.16 to 1.2	0.116
**Gastroenteritis**					
Follow ups ≥26 w	1	NR	0.45	0.18 to 1.09	0.077
Follow ups <26 w	4	0(0.424)	0.31	0.12 to 0.79	0.014
**Musculoskeletal disorders$**					
Follow ups ≥26 w	10	0(0.462)	1.31	0.86 to 1.24	0.699
Follow ups <26 w	18	22.6(0.187)	0.95	0.69 to 1.30	0.757
**Back pain**					
Follow ups ≥26 w	10	2.8(0.411)	1.05	0.81 to 1.33	0.713
Follow ups <26 w	16	0(0.832)	1.03	0.76 to 1.38	0.864
**Arthralgia**					
Follow ups ≥26 w	5	0(0.517)	0.84	0.59 to 1.20	0.345
Follow ups <26 w	7	0(0.317)	0.58	0.34 to 0.94	0.03
**Pain in extremity**					
Follow ups ≥26 w	2	0(0.738)	2.1	0.94 to 4.63	0.067
Follow ups <26 w	3	62.8(0.068)	1.82	0.37 to 8.86	0.457
**Gastrointestinal disorders**					

Follow ups ≥26 w	10	0(0.540)	0.96	0.79 to 1.16	0.691
Follow ups <26 w	28	27.6(0.093)	1.13	0.83 to 1.51	0.428
**Nausea**					
Follow ups ≥26 w	2	0(0.861)	0.71	0.42 to 1.18	0.192
Follow ups <26 w	7	0(0.945)	1.62	0.87 to 3.00	0.124
**Diarrhoea**					
Follow ups ≥26 w	10	0(0.804)	0.96	0.76 to 1.21	0.751
Follow ups <26 w	14	0(0.530)	0.85	0.59 to 1.21	0.368
**Constipation**					
Follow ups ≥26 w	1	NR	3.12	0.96 to 10.13	0.058
Follow ups <26 w	12	0(0.731)	1.5	0.85 to 2.62	0.155
**Osmotic diuresis-related AEs**					
Follow ups ≥26 w	14	0(0.893)	2.69	2.03 to 3.56	<0.001
Follow ups <26 w	21	0(0.993)	2.8	1.98 to 3.93	<0.001
**Pollakiuria**					
Follow ups ≥26 w	7	0(0.953)	2.47	1.35 to 4.50	0.003
Follow ups <26 w	19	0(1.000)	2.52	1.68 to 3.75	<0.001
**Volume-related AEs**					
Follow ups ≥26 w	30	35.1(0.032)	1.61	1.18 to 2.19	0.002
Follow ups <26 w	32	0(0.994)	1.19	0.82 to 1.70	0.349
**Postural dizziness or dizziness**					
Follow ups ≥26 w	10	22.6(0.235)	1.31	0.86 to 1.97	0.2
Follow ups <26 w	17	0(0.948)	0.88	0.56 to 1.35	0.545
**Renal-related adverse events**					
Follow ups ≥26 w	14	66.4(<0.001)	1.39	0.92 to 2.10	0.115
Follow ups <26 w	19	0(0.692)	1.31	0.89 to 1.91	0.16
**Blood creatinine increased#**					
Follow ups ≥26 w	6	0(0.840)	1.68	1.12 to 2.51	0.012
Follow ups <26 w	4	11.6(0.335)	2.25	0.44 to 11.45	0.33

**Renal failure**					
Follow ups ≥26 w	5	57.3(0.071)	1.16	0.43 to 3.07	0.773
**Metabolism and nutrition**					
Follow ups ≥26 w	4	12.4(0.330)	1.31	0.66 to 2.55	0.438
Follow ups <26 w	8	57.6(0.021)	0.8	0.53 to 1.20	0.295
**Dyslipidemia**					
Follow ups ≥26 w	3	32.4(0.228)	1.54	0.64 to 3.65	0.331
Follow ups <26 w	7	60.9(0.018)	0.85	0.54 to 1.33	0.49
**Hyperuricemia**					
Follow ups ≥26 w	1	NR	0.47	0.03 to 7.44	0.594
Follow ups <26 w	1	NR	0.55	0.21 to 1.38	0.202
**Other adverse events**					
**Hypoglycemia**					
Follow ups ≥26 w	32	50.4(0.001)	1.12	1.03 to 1.21	0.004
Follow ups <26 w	50	7.0(0.336)	1.34	1.18 to 1.51	<0.001
**Hypertension**					
Follow ups ≥26 w	8	5.3(0.389)	0.67	0.50 to 0.88	0.006
Follow ups <26 w	14	17.2(0.265)	0.54	0.39 to 0.73	<0.001
**Headache**					
Follow ups ≥26 w	10	0(0.471)	1.06	0.83 to 1.33	0.638
Follow ups <26 w	17	0(0.545)	0.74	0.54 to 0.99	0.046
**Fractures**					
Follow ups ≥26 w	7	0(0.443)	1.01	0.80 to 1.27	0.918
Follow ups <26 w	7	0(0.623)	0.46	0.22 to 0.96	0.039
**Hyperkalemia**					
Follow ups ≥26 w	2	0(0.374)	0.65	0.34 to 1.24	0.196
**Edema or edema peripheral**					
Follow ups ≥26 w	2	0(0.992)	0.49	0.27 to 0.87	0.016
Follow ups <26 w	2	0(0.939)	0.48	0.33 to 0.71	0.006

**Blood ketone bodies increased &**					
Follow ups ≥26 w	2	NR	1.99	0.22 to 17.80	0.538
Follow ups <26 w	6	0(0.787)	2.01	0.97 to 4.11	0.057
**Skin and tissue disorders**					
Follow ups <26 w	5	0(0.499)	1.82	0.81 to 4.06	0.145
**Cough**					
Follow ups ≥26 w	4	42.7(0.155)	1.2	0.58 to 2.45	0.614
Follow ups <26 w	3	47.4(0.150)	0.86	0.21 to 3.43	0.828
**Monotherapy or not**					
**All adverse events**					
**Any adverse events**					
monotherapy	16	0(0.608)	0.99	0.97 to 1.00	0.094
multitherapy	66	0(0.601)	1.01	0.98 to 1.02	0.498
**Serious adverse events**					
monotherapy	16	0(0.795)	0.91	0.85 to 0.95	<0.001
multitherapy	65	0(0.876)	0.87	0.78 to 0.95	<0.001
**AEs leading to discontinuation**					
monotherapy	16	3.9(0.409)	0.96	0.84 to 1.07	0.466
multitherapy	62	2.4(0.424)	1.02	0.89 to 1.17	0.741
**AEs related to studied drugs**					
monotherapy	12	52.2(0.018)	1.26	1.10 to 1.43	0.001
multitherapy	43	36.8(0.010)	1.37	1.25 to 1.50	<0.001
**Death**					
monotherapy	13	0(0.795)	0.76	0.61 to 0.94	0.013
multitherapy	43	0(0.991)	0.84	0.51 to 1.35	0.474
**Adverse events occurring in ≥3% patients or other clinical interest**					
**Infections and infestations**					
monotherapy	16	67.1(<0.001)	1.3	1.11 to 1.51	0.001
multitherapy	67	54.3(<0.001)	1.29	1.17 to 1.41	<0.001

**Urinary tract infection (UTI)**					
monotherapy	16	0(0.686)	1.01	0.92 to 1.10	0.834
multitherapy	62	0(0.904)	1.09	0.98 to 1.20	0.107
**Genital mycotic infection (GMI)**					
monotherapy	16	19.1(0.235)	3.48	2.54 to 4.76	<0.001
multitherapy	57	0(0.630)	3.87	3.17 to 4.71	<0.001
**Respiratory tract infection***					
monotherapy	1	NR	1.49	0.94 to 2.36	0.088
multitherapy	26	2.8(0.422)	0.92	0.76 to 1.10	0.109
**Bronchitis**					
monotherapy	1	NR	0.75	0.37 to 1.52	0.431
multitherapy	11	20.0(0.253)	1.13	0.77 to 1.64	0.533
**Nasopharyngitis**					
monotherapy	6	0(0.804)	0.81	0.64 to 1.00	0.054
multitherapy	38	0(0.826)	0.98	0.86 to 1.04	0.745
**Influenza**					
monotherapy	1	NR	0.88	0.44 to 1.74	0.709
multitherapy	9	50.4(0.041)	1.09	0.66 to 1.78	0.723
**Gastroenteritis**					
monotherapy	1	NR	0.45	0.18 to 1.09	0.077
multitherapy	4	0(0.424)	0.31	0.12 to 0.79	0.014
**Musculoskeletal disorders$**					
monotherapy	4	0(0.494)	1.15	0.88 to 1.48	0.297
multitherapy	24	14.9(0.255)	0.93	0.75 to 1.14	0.477
**Back pain**					
monotherapy	4	0(0.544)	1.18	0.83 to 1.66	0.349
multitherapy	22	0(0.769)	0.99	0.78 to 1.23	0.9
**Arthralgia**					
monotherapy	2	54.9(0.136)	1	0.50 to 1.97	0.996

multitherapy	10	0(0.615)	0.61	0.43 to 0.85	0.004
**Pain in extremity**					
monotherapy	1	NR	2.54	0.70 to 9.14	0.154
multitherapy	4	42.7(0.155)	1.85	0.63 to 5.33	0.258
**Gastrointestinal disorders**					
monotherapy	5	44.1(0.128)	1.13	0.75 to 1.69	0.544
multitherapy	33	16.1(0.213)	2.71	0.81 to 1.22	0.958
**Nausea**					
monotherapy	2	19.9(0.264)	1.63	0.71 to 3.72	0.244
multitherapy	7	0(0.936)	0.8	0.50 to 1.28	0.36
**Diarrhoea**					
monotherapy	5	0(0.536)	0.94	0.66 to 1.32	0.726
multitherapy	19	0(0.699)	0.92	0.72 to 1.16	0.501
**Constipation**					
monotherapy	1	NR	2.53	0.29 to 21.42	0.395
multitherapy	12	0(0.623)	1.68	0.99 to 2.82	0.051
**Osmotic diuresis-related AEs**					
monotherapy	8	0(0.882)	2.44	1.67 to 3.55	<0.001
multitherapy	27	0(0.994)	2.89	2.21 to 3.77	<0.001
**Pollakiuria**					
monotherapy	4	0(0.781)	2.13	1.19 to 3.78	0.01
multitherapy	22	0(1.000)	2.72	1.80 to 4.08	<0.001
**Volume-related AEs**					
monotherapy	15	46.1(0.026)	2.02	1.26 to 3.23	0.003
multitherapy	47	0(0.898)	1.29	1.00 to 1.64	0.042
**Postural dizziness or dizziness**					
monotherapy	6	22.7(0.263)	1.63	0.87 to 3.02	0.124
multitherapy	21	0(0.913)	0.96	0.70 to 1.30	0.792
**Renal-related adverse events**					

monotherapy	5	73.1(0.005)	1.11	0.62 to 1.97	0.715
multitherapy	28	0(0.554)	1.57	1.20 to 2.02	0.001
**Blood creatinine increased#**					
monotherapy	2	0(0.371)	1.52	0.91 to 2.51	0.105
multitherapy	8	0(0.753)	2.06	1.10 to 3.83	0.023
**Renal failure**					
monotherapy	3	59.6(0.084)	0.97	0.37 to 2.51	0.942
multitherapy	2	NR	1.16	0.43 to 3.07	0.197
**Metabolism and nutrition**					
multitherapy	12	48.4(0.030)	0.9	0.63 to 1.27	0.56
**Dyslipidemia**					
multitherapy	10	53.4(0.023)	0.97	0.66 to 1.41	0.874
**Hyperuricemia**					
multitherapy	2	0(0.923)	0.54	0.22 to 1.29	0.168
**Other adverse events**					
**Hypoglycemia**					
monotherapy	16	28.5(0.137)	1	0.96 to 1.11	0.338
multitherapy	66	21.2(0.075)	1.3	1.19 to 1.43	<0.001
**Hypertension**					
monotherapy	3	0(0.748)	0.64	0.41 to 1.00	0.053
multitherapy	19	23.5(0.172)	0.57	0.44 to 0.73	<0.001
**Headache**					
monotherapy	4	52.5(0.097)	1.08	0.60 to 1.93	0.8
multitherapy	23	0(0.638)	0.87	0.70 to 1.07	0.193
**Fractures**					
monotherapy	3	74.7(0.019)	0.93	0.27 to 3.12	0.899
multitherapy	11	0(0.801)	0.97	0.56 to 1.64	0.902
**Hyperkalemia**					
monotherapy	1	NR	0.69	0.35 to 1.34	0.277

multitherapy	1	NR	0.16	0.01 to 3.84	0.258
**Edema or edema peripheral**					
multitherapy	4	0(1.000)	0.49	0.33 to 0.71	<0.001
**Blood ketone bodies increased &**					
monotherapy	1	NR	1.99	0.22 to 17.80	0.538
multitherapy	7	0(0.787)	2.01	0.97 to 4.11	0.057
**Skin and tissue disorders**					
multitherapy	5	0(0.499)	1.82	0.81 to 4.06	0.145
**Cough**					
monotherapy	2	62.6(0.102)	0.75	0.13 to 4.27	0.747
multitherapy	5	26.7(0.244)	1.22	0.64 to 2.29	0.545

Supplementary TABLE 5. Sensitivity analysis for main safety outcomes

Study omitted	RR	95%CI	Study omitted	RR	95%CI
**Any adverse events**			**AEs related to studied drugs**		
Bode, B 2013	.994	.983 to 1.005	Bode, B 2013	1.347	1.246 to 1.456
Bode, B 2015	.993	.982 to 1.004	Bode, B 2015	1.345	1.243 to 1.456
Inagaki, N 2013	.994	.984 to 1.005	Inagaki, N 2013	1.337	1.239 to 1.444
Inagaki, N 2014	.994	.984 to 1.005	Inagaki, N 2014	1.332	1.233 to 1.439
Inagaki N 2016	.994	.984 to 1.005	Inagaki N 2016	1.337	1.239 to 1.444
Ji, L 2015	.994	.984 to 1.005	Ji, L 2015	1.341	1.240 to 1.450
Kadowaki, T 2017	.994	.984 to 1.000	Kadowaki, T 2017	1.341	1.242 to 1.448
Neal, B 2015	.993	.982 to 1.003	Neal, B 2015	1.319	1.224 to 1.422
Qiu, R 2014	.994	.984 to 1.000	Qiu, R 2014	1.331	1.234 to 1.436
Rodbard,H.W 2016	.994	.984 to 1.005	Rodbard, HW 2016	1.338	1.238 to 1.440
Stenlof, K 2013	.994	.983 to 1.004	Stenlof, K 2013	1.324	1.228 to 1.428
Wilding, JP 2013	.994	.983 to 1.004	Wilding, JP 2013	1.329	1.233 to 1.433
Wilding, JP 2013	.994	.984 to 1.005	Wilding, JP 2013	1.325	1.227 to 1.429
Yale, JF 2013	.994	.984 to 1.005	Yale, JF 2013	1.338	1.238 to 1.447
Yale, JF 2014	.995	.984 to 1.005	Yale, JF 2014	1.341	1.240 to 1.449
Yale JF 2017	.995	.984 to 1.005	Yale JF 2017	1.343	1.243 to 1.451
Araki, E 2016	.994	.984 to 1.005	Araki, E.2016	1.337	1.239 to 1.444
Bailey, C.J 2012	.994	.984 to 1.005	Bailey, C.J 2012	1.343	1.244 to 1.450
Bailey, CJ 2013	.994	.984 to 1.005	Bailey, CJ 2013	1.337	1.236 to 1.446
Bailey CJ 2015	.994	.984 to 1.005	Bailey CJ 2015	1.343	1.243 to 1.451

Bolinder, J 2012	.994	.984 to 1.005	Bolinder, J 2012	1.336	1.236 to 1.443
Bolinder, J 2014	.994	.984 to 1.005	Bolinder, J 2014	1.337	1.237 to 1.445
Cefalu, W. T 2015	.994	.983 to 1.005	Cefalu, WT2015	1.338	1.236 to 1.447
Jabbour, S. A 2014	.994	.983 to 1.005	Jabbour, SA 2014	1.337	1.239 to 1.444
Jabbour, S. A 2014	.994	.983 to 1.004	Jabbour, SA 2014	1.337	1.239 to 1.444
Ji, L 2014	.994	.984 to 1.005	Ji, L 2014	1.337	1.239 to 1.444
Kaku, K 2013	.994	.984 to 1.005	Kaku, K 2013	1.337	1.239 to 1.444
Kaku, K 2014	.994	.984 to 1.005	Kaku, K 2014	1.337	1.239 to 1.444
Kohan, DE 2014	.994	.983 to 1.004	Kohan, DE 2014	1.348	1.248 to1.456
Leiter, LA2014	.993	.982 to 1.004	Leiter, LA 2014	1.324	1.226 to 1.430
List, JF 2009	.994	.984 to 1.005	List, J. F 2009	1.337	1.239 to 1.444
Mathieu, C 2015	.994	.984 to 1.005	Mathieu, C 2015	1.337	1.239 to 1.444
Mathieu, C 2016	.995	.984 to 1.005	Mathieu, C 2016	1.337	1.239 to 1.444
Matthaei, S 2015	.994	.984 to 1.005	Matthaei, S 2015	1.337	1.239 to 1.444
Matthaei, S 2015	.994	.984 to 1.005	Matthaei, S 2015	1.337	1.239 to 1.444
Rosenstock, J 2012	.994	.984 to 1.005	Rosenstock, J 2012	1.337	1.239 to 1.444
Schumm-Draeger, P. M.2015	.994	.983 to 1.005	Schumm-Draeger, P. M. 2015	1.352	1.255 to 1.456
Stroje K 2011	.994	.984 to 1.005	Stroje K 2011	1.332	1.234 to 1.438
Stroje K 2014	.994	.984 to 1.005	Stroje K 2014	1.335	1.236 to 1.443
Weber, M. A 2016	.994	.984 to 1.005	Weber, MA 2016	1.337	1.239 to 1.444
Wilding, J.P 2012	.994	.984 to 1.005	Wilding, JP 2012	1.340	1.238 to 1.449
Wilding, J. P 2014	.994	.983 to 1.005	Wilding, JP 2014	1.339	1.238 to 1.449
Yang W 2016	.994	.984 to 1.005	Yang W 2016	1.337	1.239 to 1.444

Barnett, A. H.2014	.994	.984 to 1.005	Barnett, A. H. 2014	1.343	1.241 to 1.453
Ferrannini, E 2013	.994	.984 to 1.005	Ferrannini, E 2013	1.337	1.239 to 1.444
Haering, HU.2015	.994	.983 to 1.005	Haering, HU 2015	1.340	1.238 to 1.450
Haring, HU2013	.994	.983 to 1.004	Haring, HU 2013	1.335	1.234 to 1.444
Haring, HU.2014	.995	.984 to 1.005	Haring, HU 2014	1.341	1.240 to 1.440
Kadowaki, T 2014	.995	.984 to 1.005	Kadowaki, T 2014	1.338	1.238 to 1.445
Kovacs, CS 2014	.995	.984 to 1.005	Kovacs, CS 2014	1.349	1.250 to 1.456
Merker, L 2015	.995	.984 to 1.005	Merker, L 2015	1.344	1.243 to 1.453
Roden, M 2013	.995	.984 to 1.005	Roden, M 2013	1.329	1.230 to 1.435
Roden, M 2015	.994	.983 to 1.005	Roden, M 2015	1.335	1.235 to 1.444
Rosenstock, J 2013	.994	.984 to 1.005	Rosenstock, J 2013	1.337	1.239 to 1.444
Rosenstock, J 2014	.996	.985 to 1.000	Rosenstock, J 2014	1.349	1.249 to 1.457
Rosenstock, J 2015	.994	.984 to 1.005	Rosenstock, J 2015	1.338	1.236 to 1.44
Ross, S 2015	.994	.984 to 1.005	Ross, S 2015	1.337	1.237 to 1.445
Softeland, E 2017	.995	.985 to 1.006	Softeland, E. 2017	1.336	1.236 to 1.443
Tikkanen, I 2016	.995	.984 to 1.005	Tikkanen, I 2016	1.319	1.225 to 1.421
Zinman, B 2016	1.00	.989 to 1.018	Zinman, B 2016	1.337	1.239 to 1.444
Fonseca, VA 2013	.995	.984 to 1.005	Fonseca, V. A 2013	1.350	1.253 to 1.455
Ishihara, H 2016	.994	.983 to 1.004	Ishihara, H 2016	1.327	1.229 to 1.433
Kashiwagi, A 2014	.994	.984 to 1.005	Kashiwagi, A 2014	1.337	1.239 to 1.444
Kashiwagi,A.2015A	.995	.984 to 1.005	Kashiwagi,A 2015A	1.337	1.239 to 1.444
Kashiwagi,A.2015B	.994	.983 to 1.005	Kashiwagi,A 2015B	1.337	1.239 to 1.444
Kashiwagi,A.2015C	.994	.984 to 1.005	Kashiwagi, A 2015C	1.335	1.236 to 1.442

Lu, C.H 2016	.994	.984 to 1.005	Lu, C.H 2016	1.343	1.243 to 1.451
Wilding, J. P 2013	.994	.984 to 1.005	Wilding, JP 2013	1.336	1.236 to 1.443
Ikeda, S.2015	.994	.984 to 1.005	Ikeda, S 2015	1.337	1.239 to 1.444
Kaku, K 2014	.994	.984 to 1.005	Kaku, K 2014	1.337	1.239 to 1.444
Terauchi, Y.2017	.994	.984 to 1.005	Terauchi, Y 2017	1.331	1.232 to 1.434
SeinoY 2014A	.994	.984 to 1.005	SeinoY 2014A	1.337	1.239 to 1.444
SeinoY 2014B	.994	.984 to 1.005	SeinoY 2014B	1.337	1.239 to 1.444
SeinoY 2014C	.994	.984 to 1.005	SeinoY 2014C	1.337	1.239 to 1.444
SeinoY2015	.994	.984 to 1.005	SeinoY 2015	1.337	1.239 to 1.444
SeinoY 2018	.994	.984 to 1.005	SeinoY 2018	1.337	1.239 to 1.444
Amin NB 2015	.994	.984 to 1.005	Amin NB 2015	1.337	1.238 to 1.445
Terra, S. G.2017	.994	.984 to 1.005	Terra, SG 2017	1.334	1.234 to 1.442
Dagogo-Jack, S.2018	.995	.984 to 1.005	Dagogo-Jack, S 2018	1.336	1.236 to 1.444
Dagogo-Jack, S.201	.995	.984 to 1.005	Dagogo-Jack, S 2018	1.342	1.242 to 1.450
Grunberger, G2018	.995	.984 to 1.005	Grunberger, G 2018	1.339	1.239 to1.448
Grunberger, G2018	.994	.984 to 1.005	Grunberger, G 2018	1.341	1.240 to 1.450
Rosenstock, J.2018	.994	.984 to 1.005	Rosenstock, J 2018	1.331	1.232 to 1.438
**Serious adverse events**			**Death**		
Bode, B 2013	.897	.856 to .941	Bode, B 2013	.775	.638 to .941
Bode, B 2015	.895	.853 to .938	Bode, B 2015	.771	.635 to .937
Inagaki, N 2013	.897	.855 to .940	Inagaki, N 2013	.775	.638 to .941
Inagaki, N 2014	.897	.856 to .940	Inagaki, N 2014	.775	.638 to .941
Inagaki N 2016	.896	.855 to .940	Inagaki N 2016	.775	.638 to .941

Ji, L 2015	.896	.855 to .940	Ji, L 2015	.775	.638 to .941
Kadowaki, T 2017	.897	.855 to .940	Kadowaki, T 2017	.775	.638 to .941
Neal, B 2015	.899	.856 to .943	Neal, B 2015	.783	.642 to .956
Qiu, R 2014	.897	.855 to .940	Qiu, R 2014	.773	.637 to .939
Rodbard, H.W 2016	.897	.855 to .940	Rodbard, H.W 2016	.775	.638 to .941
Stenlof, K 2013	.896	.855 to .940	Stenlof, K 2013	.777	.640 to .944
Wilding, JP 2013	.898	.856 to .941	Wilding, JP 2013	.775	.638 to .941
Wilding, JP 2013	.898	.857 to .942	Wilding, JP 2013	.775	.638 to .941
Yale, JF 2013	.899	.857 to .942	Yale, JF 2013	.777	.640 to .943
Yale, JF 2014	.898	.856 to .941	Yale, JF 2014	.772	.635 to .939
Yale JF 2017	.898	.857 to .942	Yale JF 2017	.779	.641 to .947
Araki, E 2016	.896	.855 to .940	Araki, E 2016	.775	.638 to .941
Bailey, CJ 2012	.896	.855 to .940	Bailey, C.J 2012	.775	.638 to .941
Bailey, CJ 2013	.897	.855 to .940	Bailey, CJ 2013	.776	.639 to .942
Bailey CJ 2015	.897	.855 to .940	Bailey CJ 2015	.774	.637 to .940
Bolinder, J 2012	.896	.854 to .939	Bolinder, J 2012	.771	.635 to .937
Bolinder, J 2014	.896	.854 to .939	Bolinder, J 2014	.771	.635 to .937
Cefalu, WT 2015	.893	.852 to .937	Cefalu, WT 2015	.757	.623 to .920
Jabbour, SA 2014	.896	.855 to .940	Jabbour, SA 2014	.778	.640 to .944
Jabbour, SA 2014	.897	.855 to .941	Jabbour, SA 2014	.778	.640 to .944
Ji, L 2014	.896	.854 to .939	Ji, L 2014	.775	.638 to .941
Kaku, K 2013	.896	.855 to .940	Kaku, K 2013	.775	.638 to .941
Kaku, K 2014	.897	.855 to .940	Kaku, K 2014	.775	.638 to .941

Kohan, DE 2014	.897	.855 to .940	Kohan, DE 2014	.784	.644 to .954
Leiter, LA 2014	.897	.855 to .941	Leiter, LA 2014	.767	.630 to .933
List, J. F 2009	.897	.855 to .940	List, JF 2009	.775	.638 to .941
Mathieu, C 2015	.896	.855 to .940	Mathieu, C 2015	.775	.638 to .941
Mathieu, C 2016	.896	.854 to .939	Mathieu, C 2016	.771	.635 to .937
Matthaei, S 2015	.897	.856 to .941	Matthaei, S 2015	.775	.638 to .941
Matthaei, S 2015	.897	.855 to .940	Matthaei, S 2015	.775	.638 to .941
Rosenstock, J 2012	.897	.855 to .940	Rosenstock, J 2012	.775	.638 to .941
Schumm-Draeger, P. M 2015	.896	.855 to .939	Schumm-Draeger, P. M.2015	.775	.638 to .941
Stroje K 2011	.895	.854 to .939	Stroje K 2011	.773	.636 to .938
Stroje K 2014	.895	.854 to .939	Stroje K 2014	.772	.635 to .937
Weber, M. A 2016	.896	.854 to .939	Weber, M. A 2016	.775	.638 to .941
Wilding, J.P 2012	.897	.855 to .941	Wilding, J.P 2012	.773	.636 to .938
Wilding, J. P 2014	.897	.855 to .941	Wilding, J. P 2014	.772	.635 to .937
Yang W 2016	.898	.856 to .941	Yang W 2016	.775	.638 to .941
Barnett, A. H 2014	.898	.857 to .942	Barnett, A. H. 2014	.782	.643 to .949
Ferrannini, E 2013	.896	.855 to .940	Ferrannini, E 2013	.775	.638 to .941
Haering, H.U 2015	.897	.855 to .941	Haering, HU 2015	.773	.637 to .939
Haring, H.U 2013	.899	.858 to .943	Haring, HU 2013	.773	.637 to .939
Haring, H.U 2014	.897	.855 to .940	Haring, HU 2014	.775	.638 to .941
Kadowaki, T 2014	.898	.856 to .941	Kadowaki, T 2014	.775	.638 to .941
Kovacs, CS 2014	.897	.855 to .940	Kovacs, C.S 2014	.774	.637 to .940
Merker, L 2015	.899	.857 to .942	Merker, L 2015	.775	.638 to .941

Roden, M 2013	.896	.855 to .940	Roden, M 2013	.779	.642 to .947
Roden, M 2015	.897	.855 to .940	Roden, M 2015	.779	.642 to .947
Rosenstock, J 2013	.897	.855 to .940	Rosenstock, J 2013	.775	.638 to .941
Rosenstock, J 2014	.896	.855 to .940	Rosenstock, J 2014	.773	.637 to .939
Rosenstock, J 2015	.895	.853 to .939	Rosenstock, J 2015	.779	.642 to .946
Ross, S 2015	.896	.855 to .940	Ross, S 2015	.775	.638 to .941
Softeland, E. 2017	.899	.857 to .942	Softeland, E 2017	.775	.638 to .941
Tikkanen, I 2016	.898	.856 to .941	Tikkanen, I 2016	.773	.637 to .939
Zinman, B 2016	.890	.824 to .962	Zinman, B 2016	.891	.615 to 1.291
Fonseca, V. A 2013	.897	.855 to .940	Fonseca, V.A 2013	.775	.638 to .941
Ishihara, H 2016	.897	.856 to .941	Ishihara, H 2016	.775	.638 to .941
Kashiwagi, A 2014	.898	.856 to .941	Kashiwagi, A 2014	.775	.638 to .941
Kashiwagi,A 2015A	.897	.856 to .941	Kashiwagi, A 2015A	.775	.638 to .941
Kashiwagi,A 2015B	.896	.855 to .940	Kashiwagi, A 2015B	.775	.638 to .941
Kashiwagi,A 2015C	.897	.855 to .940	Kashiwagi, A 2015C	.775	.638 to .941
Lu, C.H 2016	.897	.856 to .941	Lu, C.H 2016	.775	.638 to .941
Wilding, J. P 2013	.897	.855 to .940	Wilding, JP 2013	.775	.638 to .941
Ikeda, S 2015	.897	.855 to .940	Ikeda, S 2015	.775	.638 to .941
Kaku, K 2014	.897	.855 to .940	Kaku, K 2014	.775	.638 to .941
Terauchi, Y 2017	.898	.856 to .941	Terauchi, Y 2017	.775	.638 to .941
SeinoY 2014A	.897	.855 to .940	SeinoY 2014A	.775	.638 to .941
SeinoY 2014B	.897	.855 to .940	SeinoY 2014B	.775	.638 to .941
SeinoY 2014C	.896	.855 to .940	SeinoY 2014C	.775	.638 to .941

SeinoY 2015	.896	.855 to .940	SeinoY 2015	.775	.638 to .941
SeinoY 2018	.897	.856 to .941	SeinoY 2018	.775	.638 to .941
Amin NB 2015	.897	.855 to .940	Amin NB 2015	.775	.638 to .941
Terra, SG 2017	.896	.854 to .939	Terra, SG 2017	.775	.638 to .941
Dagogo-Jack, S 2018	.897	.855 to .940	Dagogo-Jack, S 2018	.775	.638 to .941
Dagogo-Jack, S 2018	.896	.855 to .940	Dagogo-Jack, S 2018	.775	.638 to .941
Grunberger, G 2018	.896	.855 to .940	Grunberger, G 2018	.771	.635 to .937
Grunberger, G 2018	.894	.853 to .938	Grunberger, G 2018	.769	.632 to .935
Rosenstock, J 2018	.898	.856 to .941	Rosenstock, J 2018	.775	.638 to .941
**AEs leading to discontinuation**					
Bode, B 2013	.997	.901 to 1.102	Yang W 2016	.999	.904 to 1.103
Bode, B 2015	.996	.900 to 1.101	Barnett, AH. 2014	.995	.900 to 1.100
Inagaki, N 2013	.995	.902 to 1.090	Ferrannini, E 2013	.993	.902 to 1.093
Inagaki, N 2014	.999	.904 to 1.104	Haering, HU 2015	1.003	.907 to 1.108
Inagaki N 2016	.995	.902 to 1.098	Haring, HU 2013	1.001	.905 to 1.106
Ji, L 2015	.983	.896 to 1.079	Haring, HU 2014	.993	.905 to 1.090
Kadowaki, T 2017	.999	.904 to 1.103	Kadowaki, T 2014	.965	.894 to 1.041
Neal, B 2015	.989	.894 to 1.093	Kovacs, CS 2014	.999	.904 to 1.104
Qiu, R 2014	.978	.896 to 1.068	Merker, L 2015	1.000	.905 to 1.106
Rodbard, H.W 2016	.996	.904 to 1.097	Roden, M 2013	.978	.899 to 1.063
Stenlof, K 2013	.986	.898 to 1.082	Roden, M 2015	1.001	.908 to 1.103
Wilding, JP 2013	.997	.903 to 1.101	Rosenstock, J 2013	.992	.901 to 1.092
Wilding, JP 2013	.982	.894 to 1.078	Rosenstock, J 2014	.997	.902 to 1.103

Yale, JF 2013	.999	.906 to 1.102	Rosenstock, J 2015	.967	.886 to 1.054
Yale, JF 2014	1.000	.905 to 1.105	Ross, S 2015	.988	.899 to 1.086
Yale JF 2017	.984	.898 to 1.079	Softeland, E 2017	.998	.905 to 1.101
Araki, E 2016	.998	.904 to 1.102	Tikkanen, I 2016	.999	.904 to 1.104
Bailey, C.J 2012	.997	.903 to 1.101	Zinman, B 2016	1.041	.932 to 1.163
Bailey, CJ 2013	1.000	.907 to 1.103	Fonseca, VA 2013	.993	.902 to 1.093
Bailey CJ 2015	.996	.902 to 1.100	Ishihara, H 2016	.998	.905 to 1.101
Bolinder, J 2012	.979	.896 to 1.069	Kashiwagi, A 2014	.993	.902 to 1.093
Bolinder, J 2014	.953	.883 to 1.028	Kashiwagi,A 2015A	.964	.894 to 1.040
Cefalu, WT 2015	.942	.872 to 1.018	Kashiwagi,A 2015B	.997	.902 to 1.101
Jabbour, SA 2014	.994	.901 to 1.097	Kashiwagi,A 2015C	.993	.904 to 1.090
Jabbour, SA 2014	.998	.903 to 1.104	Lu, C.H 2016	.996	.902 to 1.099
Ji, L 2014	.989	.900 to 1.088	Wilding, JP 2013	.993	.902 to 1.093
Kaku, K 2013	.997	.903 to 1.101	Ikeda, S 2015	.996	.905 to 1.096
Kaku, K 2014	.999	.903 to 1.104	Kaku, K 2014	.998	.904 to 1.103
Kohan, DE 2014	.978	.902 to 1.061	Terauchi, Y 2017	.993	.901 to 1.094
Leiter, L. A 2014	.987	.894 to 1.091	SeinoY 2014A	.996	.902 to 1.099
List, J. F 2009	.996	.903 to 1.100	SeinoY 2014B	.999	.904 to 1.103
Mathieu, C 2015	.959	.887 to 1.037	SeinoY 2014C	.993	.902 to 1.093
Mathieu, C 2016	.966	.889 to 1.048	SeinoY 2015	.998	.905 to 1.100
Matthaei, S 2015	.999	.905 to 1.103	SeinoY 2018	.962	.891 to 1.037
Matthaei, S 2015	.998	.905 to 1.100	Amin NB 2015	.996	.902 to 1.099
Rosenstock, J 2012	.993	.902 to 1.093	Terra, SG 2017	1.000	.906 to 1.104

Schumm-Draeger, P. M 2015	.991	.898 to 1.093	Dagogo-Jack, S 2018	.990	.900 to 1.089
Stroje K 2011	.993	.900 to 1.096	Dagogo-Jack, S 2018	.998	.903 to 1.103
Stroje K 2014	.999	.904 to 1.105	Grunberger, G 2018	.996	.902 to 1.101
Weber, MA 2016	.992	.903 to 1.090	Grunberger, G 2018	.995	.901 to 1.100
Wilding, JP 2012	.995	.900 to 1.100	Rosenstock, J 2018	.998	.903 to 1.103
Wilding, JP 2014	.998	.902 to 1.104			

Supplementary TABLE S6 Further researches of relative risks of adverse events reported for SGLT2 inhibitors in comparison to Placebo in 70 researches

	No. of studies	I^2(P value)	RR	95%CI	P
**All adverse events**					
**Any adverse events**	69	36.9(0.002)	0.99	0.97-1.01	0.523
**Serious adverse events**	68	0(0.985)	0.90	0.86-0.95	< 0.001
**AEs leading to discontinuation**	65	4.7(0.371)	0.98	0.88-1.09	0.730
**AEs related to studied drugs**	45	55.2(0.000)	1.31	1.20-1.44	< 0.001
**Death**	44	0(0.975)	0.77	0.63-0.94	0.009
**Adverse events occurring in ≥3% patients or other clinical interest**					
**Infections and infestations**	69	62.4(0.000)	1.21	1.11-1.32	<0.001
**Urinary tract infection(UTI)**	65	0(0.850)	1.03	0.96-1.10	0.437
**Genital mycotic infection(GMI)**	60	3.9(0.390)	3.47	2.93-4.12	< 0.001
**Respiratory tract infection***	24	17.9(0.216)	0.94	0.77-1.15	0.545
**Bronchitis**	10	4.1(0.403)	0.95	0.70-1.28	0.714
**Nasopharyngitis**	38	0(0.817)	0.92	0.83-1.02	0.119
**Influenza**	9	56.2(0.019)	1.02	0.61-1.71	0.935
**Gastroenteritis**	5	0(0.546)	0.38	0.20-0.72	0.003
**Musculoskeletal disorders$**					
**Overall**	24	7.2(0.362)	1.05	0.89-1.24	0.564
**Back pain**	23	0(0.692)	1.05	0.86-1.28	0.634
**Arthralgia**	9	0(0.460)	0.82	0.61-1.10	0.187
**Pain in extremity**	5	25.5(0.252)	1.99	0.93-4.23	0.075
**Gastrointestinal disorders**					
**Overall**	31	0(0.569)	1.03	0.88-1.21	0.681
**Nausea**	9	0(0.663)	1.00	0.67-1.48	0.984
**Diarrhoea**	20	0(0.873)	0.97	0.79-1.19	0.762
**Constipation**	12	0(0.674)	1.55	0.89-2.72	0.121

**Osmotic diuresis-related AEs**					
**Overall**	28	0(1.000)	2.67	2.09-3.41	< 0.001
**Pollakiuria**	24	0(1.000)	2.61	1.82-3.73	< 0.001
**Volume-related AEs**					
**Overall**	47	0(0.728)	1.29	1.10-1.51	0.002
**Postural dizziness or dizziness**	22	0(0.732)	1.19	0.91-1.56	0.212
**Renal-related adverse events**					
**Overall**	22	51.5(0.004)	1.40	1.00-1.97	0.050
**Blood creatinine increased#**	8	0(0.710)	1.65	1.11-2.46	0.014
**Renal failure**	5	57.3(0.071)	1.16	0.43-3.08	0.773
**Metabolism and nutrition**					
**Overall**	11	51.9(0.023)	0.87	0.59-1.28	0.480
**Dyslipidemia**	9	58.0(0.015)	0.94	0.61-1.45	0.786
**Hyperuricemia**	2	0(0.923)	0.54	0.22-1.30	0.168
**Other adverse events**					
**Hypoglycemia**	69	31.9(0.009)	1.14	1.07-1.23	< 0.001
**Hypertension**	18	22.3(0.195)	0.59	0.45-0.76	< 0.001
**Headache**	24	7.9(0.352)	0.96	0.78-1.19	0.721
**Fractures**	14	9.6(0.350)	0.90	0.64-1.27	0.552
**Hyperkalemia**	2	0(0.374)	0.65	0.34-1.25	0.196
**Edema or edema peripheral**	3	0(1.000)	0.49	0.31-0.80	0.004
**Blood ketone bodies increased &**	8	0(0.877)	2.01	1.01-3.97	0.046
**Skin and tissue disorders**	5	0(0.499)	1.82	0.81-4.06	0.145
**Cough**	7	53.8(0.043)	1.02	0.53-1.97	0.952

Supplementary FIGURE S1

Flow diagram for the selection of eligible randomized controlled trials. RCT indicates randomized controlled trial.

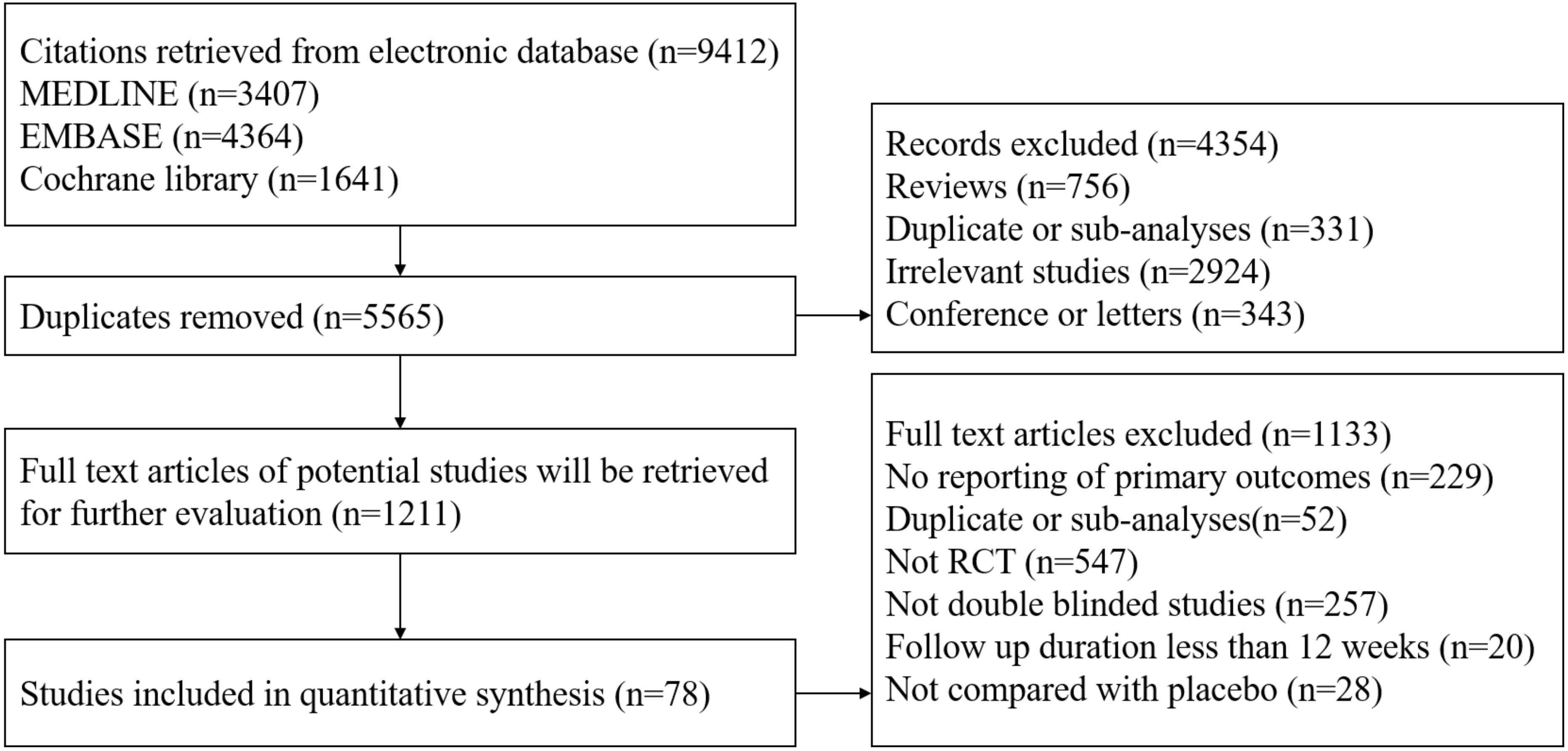

Supplementary FIGURE S2 Funnel plot of main safety indexes

A

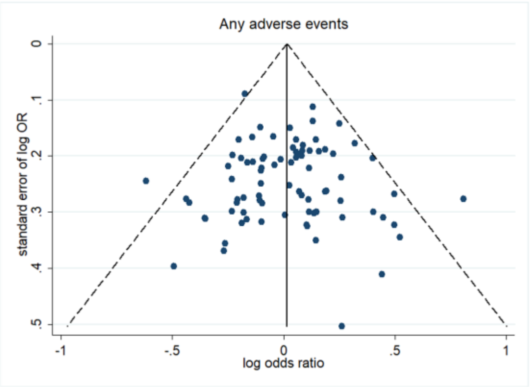

B

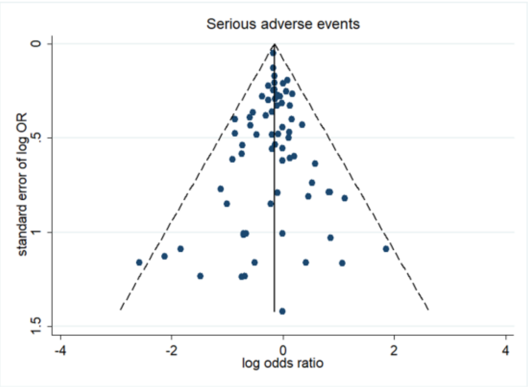

P= 0.671(Begg's Test) and *P*=0.744 (Egger's Test)

P=0.517 (Begg's Test) and *P*= 0.739(Egger's Test)

C

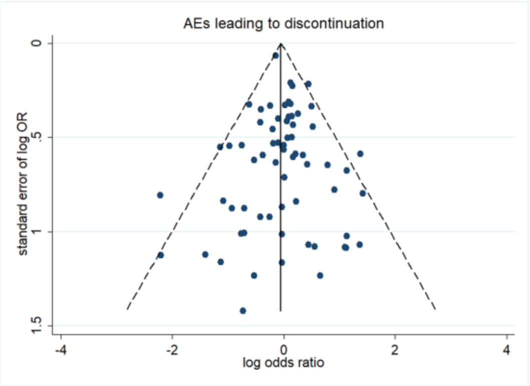

D

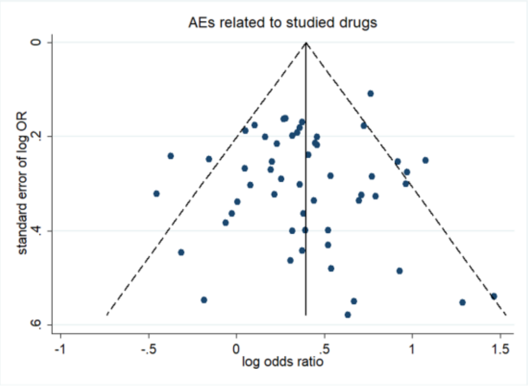

P=0.312 (Begg's Test) and *P*=0.637 (Egger's Test)

P=0.257 (Begg's Test) and *P*=0.761 (Egger's Test)

E

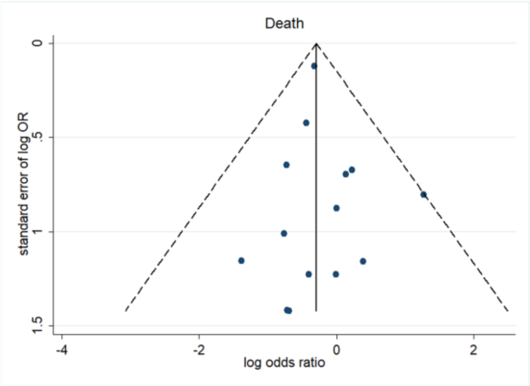

P=0.913 (Begg's Test) and *P*=0.596 (Egger's Test)

References

1. Bode B, Stenlof K, Harris S, et al. Long-term efficacy and safety of canagliflozin over 104 weeks in patients aged 55-80 years with type 2 diabetes. Diabetes Obes Metab. 2015;17:294-303. doi: 10.1111/dom.12428. Epub 2015 Jan 12.
2. Bode B, Stenlof K, Sullivan D, et al. Efficacy and safety of canagliflozin treatment in older subjects with type 2 diabetes mellitus: a randomized trial. 1995). 2013;41:72-84. doi: 10.3810/hp.2013.04.1020.
3. Inagaki N, Harashima S, Maruyama N, et al. Efficacy and safety of canagliflozin in combination with insulin: a double-blind, randomized, placebo-controlled study in Japanese patients with type 2 diabetes mellitus. Cardiovasc Diabetol 2016;15:89.
4. Inagaki N, Kondo K, Yoshinari T, et al. Efficacy and safety of canagliflozin in Japanese patients with type 2 diabetes: a randomized, double-blind, placebo-controlled, 12-week study. Diabetes Obes Metab 2013;15:1136-45.
5. Inagaki N, Kondo K, Yoshinari T, et al. Efficacy and safety of canagliflozin monotherapy in Japanese patients with type 2 diabetes inadequately controlled with diet and exercise: a 24-week, randomized, double-blind, placebo-controlled, Phase III study. Expert Opin Pharmacother. 2014;15:1501-15. doi: 10.1517/14656566.2014.935764.
6. Ji L, Han P, Liu Y, et al. Canagliflozin in Asian patients with type 2 diabetes on metformin alone or metformin in combination with sulphonylurea. Diabetes Obes Metab. 2015;17:23-31. doi: 10.1111/dom.12385. Epub 2014 Oct 14.
7. Kadowaki T, Inagaki N, Kondo K, et al. Efficacy and safety of canagliflozin as add-on therapy to teneligliptin in Japanese patients with type 2 diabetes mellitus: Results of a 24-week, randomized, double-blind, placebo-controlled trial. Diabetes Obes Metab. 2017;19:874-882. doi: 10.1111/dom.12898. Epub 2017 Mar 31.
8. Neal B, Perkovic V, de Zeeuw D, et al. Efficacy and safety of canagliflozin, an inhibitor of sodium-glucose cotransporter 2, when used in conjunction with insulin therapy in patients with type 2 diabetes. Diabetes Care. 2015;38:403-11. doi: 10.2337/dc14-1237. Epub 2014 Dec 2.
9. Qiu R, Capuano G, Meininger G. Efficacy and safety of twice-daily treatment with canagliflozin, a sodium glucose co-transporter 2 inhibitor, added on to metformin monotherapy in patients with type 2 diabetes mellitus. Journal of Clinical & Translational Endocrinology 2014;1:54-60.
10. Rodbard HW, Seufert J, Aggarwal N, et al. Efficacy and safety of titrated canagliflozin in patients with type 2 diabetes mellitus inadequately controlled on metformin and sitagliptin. Diabetes Obes Metab 2016;18:812-9.
11. Stenlof K, Cefalu WT, Kim KA, et al. Efficacy and safety of canagliflozin monotherapy in subjects with type 2 diabetes mellitus inadequately controlled with diet and exercise. Diabetes Obes Metab. 2013;15:372-82. doi: 10.1111/dom.12054. Epub 2013 Jan 24.
12. Wilding JP, Charpentier G, Hollander P, et al. Efficacy and safety of canagliflozin in patients with type 2 diabetes mellitus inadequately controlled with metformin and sulphonylurea: a randomised trial. Int J Clin Pract. 2013;67:1267-82. doi: 10.1111/ijcp.12322. Epub 2013 Oct 13.
13. Yale JF, Bakris G, Cariou B, et al. Efficacy and safety of canagliflozin over 52 weeks in patients with type 2 diabetes mellitus and chronic kidney disease. Diabetes Obes Metab. 2014;16:1016-27. doi: 10.1111/dom.12348. Epub 2014 Jul 22.
14. Yale JF, Bakris G, Cariou B, et al. Efficacy and safety of canagliflozin in subjects with type 2 diabetes and chronic kidney disease. Diabetes Obes Metab. 2013;15:463-73. doi: 10.1111/dom.12090. Epub 2013 Mar 28.
15. Yale JF, Xie J, Sherman SE, et al. Canagliflozin in Conjunction With Sulfonylurea Maintains Glycemic Control and Weight Loss Over 52 Weeks: A Randomized, Controlled Trial in Patients With Type 2 Diabetes Mellitus. Clin Ther. 2017;39:2230-2242.e2. doi: 10.1016/j.clinthera.2017.10.003. Epub 2017 Nov 3.
16. Araki E, Onishi Y, Asano M, et al. Efficacy and safety of dapagliflozin in addition to insulin therapy in Japanese patients with type 2 diabetes: Results of the interim analysis of 16-week double-blind treatment period. J Diabetes Investig 2016;7:555-64.
17. Bailey CJ, Gross JL, Hennicken D, et al. Dapagliflozin add-on to metformin in type 2 diabetes inadequately controlled with metformin: a randomized, double-blind, placebo-controlled 102-week trial. BMC Med. 2013;11:43.:10.1186/1741-7015-11-43.
18. Bailey CJ, Iqbal N, T'Joen C, et al. Dapagliflozin monotherapy in drug-naive patients with diabetes: a randomized-controlled trial of low-dose range. Diabetes Obes Metab. 2012;14:951-9. doi: 10.1111/j.1463-1326.2012.01659.x. Epub 2012 Jul 24.
19. Bailey CJ, Morales Villegas EC, Woo V, et al. Efficacy and safety of dapagliflozin monotherapy in people with Type 2 diabetes: a randomized double-blind placebo-controlled 102-week trial. Diabet Med. 2015;32:531-41. doi: 10.1111/dme.12624. Epub 2014 Nov 22.

20. Bolinder J, Ljunggren O, Johansson L, et al. Dapagliflozin maintains glycaemic control while reducing weight and body fat mass over 2 years in patients with type 2 diabetes mellitus inadequately controlled on metformin. Diabetes Obes Metab. 2014;16:159-69. doi: 10.1111/dom.12189. Epub 2013 Aug 29.
21. Bolinder J, Ljunggren O, Kullberg J, et al. Effects of dapagliflozin on body weight, total fat mass, and regional adipose tissue distribution in patients with type 2 diabetes mellitus with inadequate glycemic control on metformin. J Clin Endocrinol Metab. 2012;97:1020-31. doi: 10.1210/jc.2011-2260. Epub 2012 Jan 11.
22. Cefalu WT, Leiter LA, de Bruin TW, et al. Dapagliflozin's Effects on Glycemia and Cardiovascular Risk Factors in High-Risk Patients With Type 2 Diabetes: A 24-Week, Multicenter, Randomized, Double-Blind, Placebo-Controlled Study With a 28-Week Extension. Diabetes Care. 2015;38:1218-27. doi: 10.2337/dc14-0315. Epub 2015 Apr 7.
23. Jabbour SA, Hardy E, Sugg J, et al. Dapagliflozin is effective as add-on therapy to sitagliptin with or without metformin: a 24-week, multicenter, randomized, double-blind, placebo-controlled study. Diabetes Care 2014;37:740-50. doi: 10.2337/dc13-0467. Epub 2013 Oct 21.
24. Ji L, Ma J, Li H, et al. Dapagliflozin as monotherapy in drug-naive Asian patients with type 2 diabetes mellitus: a randomized, blinded, prospective phase III study. Clin Ther. 2014;36:84-100.e9. doi: 10.1016/j.clinthera.2013.11.002. Epub 2013 Dec 28.
25. Kaku K, Inoue S, Matsuoka O, et al. Efficacy and safety of dapagliflozin as a monotherapy for type 2 diabetes mellitus in Japanese patients with inadequate glycaemic control: a phase II multicentre, randomized, double-blind, placebo-controlled trial. Diabetes Obes Metab. 2013;15:432-40. doi: 10.1111/dom.12047. Epub 2013 Jan 25.
26. Kaku K, Kiyosue A, Inoue S, et al. Efficacy and safety of dapagliflozin monotherapy in Japanese patients with type 2 diabetes inadequately controlled by diet and exercise. Diabetes Obes Metab. 2014;16:1102-10. doi: 10.1111/dom.12325. Epub 2014 Jul 8.
27. Kohan DE, Fioretto P, Tang W, et al. Long-term study of patients with type 2 diabetes and moderate renal impairment shows that dapagliflozin reduces weight and blood pressure but does not improve glycemic control. Kidney Int. 2014;85:962-71. doi: 10.1038/ki.2013.356. Epub 2013 Sep 25.
28. Leiter LA, Cefalu WT, de Bruin TW, et al. Dapagliflozin added to usual care in individuals with type 2 diabetes mellitus with preexisting cardiovascular disease: a 24-week, multicenter, randomized, double-blind, placebo-controlled study with a 28-week extension. J Am Geriatr Soc 2014;62:1252-62.
29. List JF, Woo V, Morales E, et al. Sodium-glucose cotransport inhibition with dapagliflozin in type 2 diabetes. Diabetes Care. 2009;32:650-7. doi: 10.2337/dc08-1863. Epub 2008 Dec 29.
30. Mathieu C, Herrera Marmolejo M, Gonzalez Gonzalez JG, et al. Efficacy and safety of triple therapy with dapagliflozin add-on to saxagliptin plus metformin over 52 weeks in patients with type 2 diabetes. Diabetes Obes Metab. 2016;18:1134-1137. doi: 10.1111/dom.12737. Epub 2016 Aug 19.
31. Mathieu C, Ranetti AE, Li D, et al. Randomized, Double-Blind, Phase 3 Trial of Triple Therapy With Dapagliflozin Add-on to Saxagliptin Plus Metformin in Type 2 Diabetes. Diabetes Care. 2015;38:2009-17. doi: 10.2337/dc15-0779. Epub 2015 Aug 5.
32. Matthaei S, Bowering K, Rohwedder K, et al. Durability and tolerability of dapagliflozin over 52 weeks as add-on to metformin and sulphonylurea in type 2 diabetes. Diabetes Obes Metab. 2015;17:1075-84. doi: 10.1111/dom.12543. Epub 2015 Sep 4.
33. Rosenstock J, Vico M, Wei L, et al. Effects of dapagliflozin, an SGLT2 inhibitor, on HbA(1c), body weight, and hypoglycemia risk in patients with type 2 diabetes inadequately controlled on pioglitazone monotherapy. Diabetes Care. 2012;35:1473-8. doi: 10.2337/dc11-1693. Epub 2012 Mar 23.
34. Schumm-Draeger PM, Burgess L, Koranyi L, et al. Twice-daily dapagliflozin co-administered with metformin in type 2 diabetes: a 16-week randomized, placebo-controlled clinical trial. Diabetes Obes Metab. 2015;17:42-51. doi: 10.1111/dom.12387. Epub 2014 Oct 16.
35. Strojek K, Yoon KH, Hruba V, et al. Effect of dapagliflozin in patients with type 2 diabetes who have inadequate glycaemic control with glimepiride: a randomized, 24-week, double-blind, placebo-controlled trial. Diabetes Obes Metab. 2011;13:928-38. doi: 10.1111/j.1463-1326.2011.01434.x.
36. Strojek K, Yoon KH, Hruba V, et al. Dapagliflozin added to glimepiride in patients with type 2 diabetes mellitus sustains glycemic control and weight loss over 48 weeks: a randomized, double-blind, parallel-group, placebo-controlled trial. Diabetes Ther. 2014;5:267-83. doi: 10.1007/s13300-014-0072-0. Epub 2014 Jun 12.
37. Weber MA, Mansfield TA, Cain VA, et al. Blood pressure and glycaemic effects of dapagliflozin versus placebo in patients with type 2 diabetes on combination antihypertensive therapy: a randomised, double-blind, placebo-controlled, phase 3 study. Lancet Diabetes Endocrinol. 2016;4:211-220. doi: 10.1016/S2213-8587(15)00417-9. Epub 2015 Nov 27.

38. Wilding JP, Woo V, Rohwedder K, et al. Dapagliflozin in patients with type 2 diabetes receiving high doses of insulin: efficacy and safety over 2 years. Diabetes Obes Metab. 2014;16:124-36. doi: 10.1111/dom.12187. Epub 2013 Aug 29.
39. Wilding JP, Woo V, Soler NG, et al. Long-term efficacy of dapagliflozin in patients with type 2 diabetes mellitus receiving high doses of insulin: a randomized trial. Ann Intern Med. 2012;156:405-15. doi: 10.7326/0003-4819-156-6-201203200-00003.
40. Yang W, Han P, Min KW, et al. Efficacy and safety of dapagliflozin in Asian patients with type 2 diabetes after metformin failure: A randomized controlled trial. Journal of Diabetes 2016.
41. Barnett AH, Mithal A, Manassie J, et al. Efficacy and safety of empagliflozin added to existing antidiabetes treatment in patients with type 2 diabetes and chronic kidney disease: a randomised, double-blind, placebo-controlled trial. Lancet Diabetes Endocrinol. 2014;2:369-84. doi: 10.1016/S2213-8587(13)70208-0. Epub 2014 Jan 24.
42. Ferrannini E, Seman L, Seewaldt-Becker E, et al. A Phase IIb, randomized, placebo-controlled study of the SGLT2 inhibitor empagliflozin in patients with type 2 diabetes. Diabetes Obes Metab. 2013;15:721-8. doi: 10.1111/dom.12081. Epub 2013 Mar 4.
43. Haering HU, Merker L, Christiansen AV, et al. Empagliflozin as add-on to metformin plus sulphonylurea in patients with type 2 diabetes. Diabetes Res Clin Pract. 2015;110:82-90. doi: 10.1016/j.diabres.2015.05.044. Epub 2015 May 29.
44. Haring HU, Merker L, Seewaldt-Becker E, et al. Empagliflozin as add-on to metformin in patients with type 2 diabetes: a 24-week, randomized, double-blind, placebo-controlled trial. Diabetes Care. 2014;37:1650-9. doi: 10.2337/dc13-2105. Epub 2014 Apr 10.
45. Haring HU, Merker L, Seewaldt-Becker E, et al. Empagliflozin as add-on to metformin plus sulfonylurea in patients with type 2 diabetes: a 24-week, randomized, double-blind, placebo-controlled trial. Diabetes Care. 2013;36:3396-404. doi: 10.2337/dc12-2673. Epub 2013 Aug 20.
46. Kadowaki T, Haneda M, Inagaki N, et al. Empagliflozin monotherapy in Japanese patients with type 2 diabetes mellitus: a randomized, 12-week, double-blind, placebo-controlled, phase II trial. Adv Ther. 2014;31:621-38. doi: 10.1007/s12325-014-0126-8. Epub 2014 Jun 24.
47. Kovacs CS, Seshiah V, Swallow R, et al. Empagliflozin improves glycaemic and weight control as add-on therapy to pioglitazone or pioglitazone plus metformin in patients with type 2 diabetes: a 24-week, randomized, placebo-controlled trial. Diabetes Obes Metab. 2014;16:147-58. doi: 10.1111/dom.12188. Epub 2013 Aug 22.
48. Merker L, Haring HU, Christiansen AV, et al. Empagliflozin as add-on to metformin in people with Type 2 diabetes. Diabet Med. 2015;32:1555-67. doi: 10.1111/dme.12814. Epub 2015 Jul 14.
49. Roden M, Merker L, Christiansen AV, et al. Safety, tolerability and effects on cardiometabolic risk factors of empagliflozin monotherapy in drug-naive patients with type 2 diabetes: a double-blind extension of a Phase III randomized controlled trial. Cardiovasc Diabetol. 2015;14:154.:10.1186/s12933-015-0314-0.
50. Roden M, Weng J, Eilbracht J, et al. Empagliflozin monotherapy with sitagliptin as an active comparator in patients with type 2 diabetes: a randomised, double-blind, placebo-controlled, phase 3 trial. Lancet Diabetes Endocrinol. 2013;1:208-19. doi: 10.1016/S2213-8587(13)70084-6. Epub 2013 Sep 9.
51. Rosenstock J, Jelaska A, Frappin G, et al. Improved glucose control with weight loss, lower insulin doses, and no increased hypoglycemia with empagliflozin added to titrated multiple daily injections of insulin in obese inadequately controlled type 2 diabetes. Diabetes Care. 2014;37:1815-23. doi: 10.2337/dc13-3055. Epub 2014 Jun 14.
52. Rosenstock J, Jelaska A, Zeller C, et al. Impact of empagliflozin added on to basal insulin in type 2 diabetes inadequately controlled on basal insulin: a 78-week randomized, double-blind, placebo-controlled trial. Diabetes Obes Metab. 2015;17:936-48. doi: 10.1111/dom.12503. Epub 2015 Jul 14.
53. Rosenstock J, Seman LJ, Jelaska A, et al. Efficacy and safety of empagliflozin, a sodium glucose cotransporter 2 (SGLT2) inhibitor, as add-on to metformin in type 2 diabetes with mild hyperglycaemia. Diabetes Obes Metab. 2013;15:1154-60. doi: 10.1111/dom.12185. Epub 2013 Aug 22.
54. Ross S, Thamer C, Cescutti J, et al. Efficacy and safety of empagliflozin twice daily versus once daily in patients with type 2 diabetes inadequately controlled on metformin: a 16-week, randomized, placebo-controlled trial. Diabetes Obes Metab 2015;17:699-702.
55. Softeland E, Meier JJ, Vangen B, et al. Empagliflozin as Add-on Therapy in Patients With Type 2 Diabetes Inadequately Controlled With Linagliptin and Metformin: A 24-Week Randomized, Double-Blind, Parallel-Group Trial. Diabetes Care. 2017;40:201-209. doi: 10.2337/dc16-1347. Epub 2016 Dec 2.
56. Tikkanen I, Narko K, Zeller C, et al. Empagliflozin reduces blood pressure in patients with type 2 diabetes and hypertension. Diabetes Care. 2015;38:420-8. doi: 10.2337/dc14-1096. Epub 2014 Sep 30.

57. Zinman B, Wanner C, Lachin JM, et al. Empagliflozin, Cardiovascular Outcomes, and Mortality in Type 2 Diabetes. N Engl J Med. 2015;373:2117-28. doi: 10.1056/NEJMoa1504720. Epub 2015 Sep 17.
58. Fonseca VA, Ferrannini E, Wilding JP, et al. Active- and placebo-controlled dose-finding study to assess the efficacy, safety, and tolerability of multiple doses of ipragliflozin in patients with type 2 diabetes mellitus. J Diabetes Complications. 2013;27:268-73. doi: 10.1016/j.jdiacomp.2012.11.005. Epub 2012 Dec 29.
59. Ishihara H, Yamaguchi S, Nakao I, et al. Efficacy and safety of ipragliflozin as add-on therapy to insulin in Japanese patients with type 2 diabetes mellitus (IOLITE): a multi-centre, randomized, placebo-controlled, double-blind study. Diabetes Obes Metab. 2016;18:1207-1216. doi: 10.1111/dom.12745. Epub 2016 Sep 15.
60. Kashiwagi A, Kazuta K, Goto K, et al. Ipragliflozin in combination with metformin for the treatment of Japanese patients with type 2 diabetes: ILLUMINATE, a randomized, double-blind, placebo-controlled study. Diabetes Obes Metab. 2015;17:304-8. doi: 10.1111/dom.12331. Epub 2014 Jul 31.
61. Kashiwagi A, Kazuta K, Takinami Y, et al. Ipragliflozin improves glycemic control in Japanese patients with type 2 diabetes mellitus: the BRIGHTEN study. Diabetology International 2015;6:8-18.
62. Kashiwagi A, Kazuta K, Yoshida S, et al. Randomized, placebo-controlled, double-blind glycemic control trial of novel sodium-dependent glucose cotransporter 2 inhibitor ipragliflozin in Japanese patients with type 2 diabetes mellitus. Journal of Diabetes Investigation 2014;5:382-391.
63. Kashiwagi A, Takahashi H, Ishikawa H, et al. A randomized, double-blind, placebo-controlled study on long-term efficacy and safety of ipragliflozin treatment in patients with type 2 diabetes mellitus and renal impairment: results of the long-term ASP1941 safety evaluation in patients with type 2 diabetes with renal impairment (LANTERN) study. Diabetes Obes Metab. 2015;17:152-60. doi: 10.1111/dom.12403.
64. Lu CH, Min KW, Chuang LM, et al. Efficacy, safety, and tolerability of ipragliflozin in Asian patients with type 2 diabetes mellitus and inadequate glycemic control with metformin: Results of a phase 3 randomized, placebo-controlled, double-blind, multicenter trial. J Diabetes Investig. 2016;7:366-73. doi: 10.1111/jdi.12422. Epub 2015 Oct 14.
65. Wilding JP, Ferrannini E, Fonseca VA, et al. Efficacy and safety of ipragliflozin in patients with type 2 diabetes inadequately controlled on metformin: a dose-finding study. Diabetes Obes Metab. 2013;15:403-9. doi: 10.1111/dom.12038. Epub 2012 Dec 7.
66. Ikeda S, Takano Y, Cynshi O, et al. A novel and selective sodium-glucose cotransporter-2 inhibitor, tofogliflozin, improves glycaemic control and lowers body weight in patients with type 2 diabetes mellitus. Diabetes Obes Metab. 2015;17:984-93. doi: 10.1111/dom.12538. Epub 2015 Aug 20.
67. Kaku K, Watada H, Iwamoto Y, et al. Efficacy and safety of monotherapy with the novel sodium/glucose cotransporter-2 inhibitor tofogliflozin in Japanese patients with type 2 diabetes mellitus: a combined Phase 2 and 3 randomized, placebo-controlled, double-blind, parallel-group comparative study. Cardiovasc Diabetol 2014;13:65.
68. Terauchi Y, Tamura M, Senda M, et al. Efficacy and safety of tofogliflozin in Japanese patients with type 2 diabetes mellitus with inadequate glycaemic control on insulin therapy (J-STEP/INS): Results of a 16-week randomized, double-blind, placebo-controlled multicentre trial. Diabetes Obes Metab 2017;19:1397-1407.
69. Seino Y, Inagaki N, Haneda M, et al. Efficacy and safety of luseogliflozin added to various oral antidiabetic drugs in Japanese patients with type 2 diabetes mellitus. J Diabetes Investig. 2015;6:443-53. doi: 10.1111/jdi.12316. Epub 2015 Jan 10.
70. Seino Y, Sasaki T, Fukatsu A, et al. Efficacy and safety of luseogliflozin added to insulin therapy in Japanese patients with type 2 diabetes: a multicenter, 52-week, clinical study with a 16-week, double-blind period and a 36-week, open-label period. Curr Med Res Opin 2018;15:1-35.
71. Seino Y, Sasaki T, Fukatsu A, et al. Efficacy and safety of luseogliflozin monotherapy in Japanese patients with type 2 diabetes mellitus: a 12-week, randomized, placebo-controlled, phase II study. Curr Med Res Opin. 2014;30:1219-30. doi: 10.1185/03007995.2014.901943. Epub 2014 Mar 19.
72. Seino Y, Sasaki T, Fukatsu A, et al. Efficacy and safety of luseogliflozin as monotherapy in Japanese patients with type 2 diabetes mellitus: a randomized, double-blind, placebo-controlled, phase 3 study. Curr Med Res Opin. 2014;30:1245-55. doi: 10.1185/03007995.2014.912983. Epub 2014 Apr 29.
73. Seino Y, Sasaki T, Fukatsu A, et al. Dose-finding study of luseogliflozin in Japanese patients with type 2 diabetes mellitus: a 12-week, randomized, double-blind, placebo-controlled, phase II study. Curr Med Res Opin. 2014;30:1231-44. doi: 10.1185/03007995.2014.909390. Epub 2014 Apr 15.
74. Amin NB, Wang X, Jain SM, et al. Dose-ranging efficacy and safety study of ertugliflozin, a sodium-glucose co-transporter 2 inhibitor, in patients with type 2 diabetes on a background of metformin. Diabetes Obes Metab. 2015;17:591-8. doi: 10.1111/dom.12460. Epub 2015 Mar 31.
75. Dagogo-Jack S, Liu J, Eldor R, et al. Efficacy and safety of the addition of ertugliflozin in patients with type 2 diabetes mellitus inadequately controlled with metformin and sitagliptin: The VERTIS SITA2 placebo-

controlled randomized study. Diabetes Obes Metab. 2018;20:530-540. doi: 10.1111/dom.13116. Epub 2017 Oct 23.

76. Grunberger G, Camp S, Johnson J, et al. Ertugliflozin in Patients with Stage 3 Chronic Kidney Disease and Type 2 Diabetes Mellitus: The VERTIS RENAL Randomized Study. Diabetes Ther. 2018;9:49-66. doi: 10.1007/s13300-017-0337-5. Epub 2017 Nov 20.

77. Rosenstock J, Frias J, Pall D, et al. Effect of ertugliflozin on glucose control, body weight, blood pressure and bone density in type 2 diabetes mellitus inadequately controlled on metformin monotherapy (VERTIS MET). Diabetes Obes Metab. 2018;20:520-529. doi: 10.1111/dom.13103. Epub 2017 Oct 2.

78. Terra SG, Focht K, Davies M, et al. Phase III, efficacy and safety study of ertugliflozin monotherapy in people with type 2 diabetes mellitus inadequately controlled with diet and exercise alone. Diabetes Obes Metab. 2017;19:721-728. doi: 10.1111/dom.12888. Epub 2017 Feb 22.
